# Bidirectional Two-Sample Mendelian Randomization Study of Immunoglobulin G N-Glycosylation and Senescence-Associated Secretory Phenotype

**DOI:** 10.3390/ijms25126337

**Published:** 2024-06-07

**Authors:** Haotian Wang, Di Liu, Xiaoni Meng, Wenxin Sun, Cancan Li, Huimin Lu, Deqiang Zheng, Lijuan Wu, Shengzhi Sun, Youxin Wang

**Affiliations:** 1 Beijing Key Laboratory of Clinical Epidemiology, School of Public Health, Capital Medical University, Beijing 100069, China; 2 Centre for Biomedical Information Technology, Shenzhen Institutes of Advanced Technology, Chinese Academy of Sciences, Shenzhen 518055, China; 3 School of Public Health, North China University of Science and Technology, Tangshan 063210, China; 4 Centre for Precision Medicine, Edith Cowan University, Perth 6027, Australia

**Keywords:** ageing, IgG N-glycosylation, Mendelian randomization, senescence-associated secretory phenotype (SASP)

## Abstract

Observational studies revealed changes in Immunoglobulin G (IgG) N-glycosylation during the aging process. However, it lacks causal insights and remains unclear in which direction causal relationships exist. The two-sample bidirectional Mendelian randomization (MR) design was adopted to explore causal associations between IgG N-glycans and the senescence-associated secretory phenotype (SASP). Inverse variance weighted (IVW) and Wald ratio methods were used as the main analyses, supplemented by sensitivity analyses. Forward MR analyses revealed causal associations between the glycan peak (GP) and SASP, including GP6 (odds ratio [OR] = 0.428, 95% confidence interval [CI] = 0.189–0.969) and GP17 (OR = 0.709, 95%CI = 0.504–0.995) with growth/differentiation factor 15 (GDF15), GP19 with an advanced glycosylation end-product-specific receptor (RAGE) (OR = 2.142, 95%  CI  = 1.384–3.316), and GP15 with matrix metalloproteinase 2 (MMP2) (OR = 1.136, 95%  CI =1.008–1.282). The reverse MR indicated that genetic liability to RAGE was associated with increased levels of GP17 (OR = 1.125, 95%  CI  = 1.003–1.261) and GP24 (OR = 1.222, 95%  CI  = 1.046–1.428), while pulmonary and activation-regulated chemokines (PARC) exhibited causal associations with GP10 (OR = 1.269, 95%  CI  = 1.048–1.537) and GP15 (OR = 1.297, 95%  CI = 1.072–1.570). The findings provided suggested evidence on the bidirectional causality between IgG N-glycans and SASP, which might reveal potential regulatory mechanisms.

## 1. Introduction

Aging is a complex process driven by multiple hallmarks [1,2]. Of these hallmarks, cellular senescence has implicitly assumed a potentially vital part in contributing to the course of the aging and age-related diseases [3,4,5]. Noteworthy, the accumulation of senescent cells causes the development of inflammatory responses. Senescent cells are permanently arrested in their cycle; however, many senescent cells change their morphology and secrete bioactive factors, including growth factors, extracellular vesicles, inflammatory cytokines, nucleotides, matrix metalloproteinases, chemokines, soluble factors, and lipids. All these factors are termed the senescence-associated secretory phenotype (SASP) [2]. Moreover, the releasing of SASP promotes cellular senescence, particularly in immunocyte senescence, leading to a compromised immune function and an inability to eliminate senescent cells and inflammatory cytokines, which conversely results in further inflammation and creates a vicious cycle. Therefore, exploring the potential mechanisms of inflammation could contribute to alleviating and preventing aging and aging-related diseases.

Glycosylation has emerged as one of the crucial post-translational modifications that manifests as the enzymatic attachment of glycans to proteins and other biomolecules. It is thought to have an influence on protein flexibility, function, and stability, and participates in practically all pathophysiological developments [6]. Immunoglobulin G (IgG) is the most abundant immunoglobulin (around 75%) in plasma, with its structure being most explored [7,8]. IgG exerts its role in the immune function in terms of two conserved N-glycosylation sites in the Fc regions that bind to distinct Fc receptors and, therefore acts as a “switch” for anti-inflammatory responses or pro-inflammatory responses [9,10,11,12,13]. Aberrant IgG N-glycosylation was accounted for by its association with age-related diseases, including dementia [14], T2DM [15], HTN [16], dyslipidemia [17], and ischemic stroke [18]. In addition, IgG glycosylation experiences significant changes during the aging process, showing a decreased level of galactosylation [19,20]. However, it remains confusing that changes in the IgG N-glycome during the aging process lead to aging, or merely aging byproducts.

Mendelian randomization (MR), an effective strategy that takes advantage of genetic variants, mostly SNPs, as instrumental variables (IVs), has recently been used to evaluate the causal relationships between exposures and outcomes [21,22]. Since genetic variants are randomly assigned to offspring during meiosis, the causal associations evaluated by MR are under little influence by issues of confounding factors and cannot be affected by reverse causation bias. Previous studies have successfully used the IgG N-glycan quantitative trait locus to identify causality linked to age-related diseases [23,24,25]. In the present study, we leveraged previously identified variants associated with IgG N-glycan and SASP from available genome-wide association studies (GWASs) to construct genetic instrumental variables and explore causal associations by the framework of MR. This work might provide evidence for the biological mechanisms of cellular senescence and their potential as targets to delay or prevent multiple age-related diseases from perspective of IgG N-glycosylation.

## 2. Results

### 2.1. Forward Associations of IgG N-Glycans with SASP

**Figure 1:**
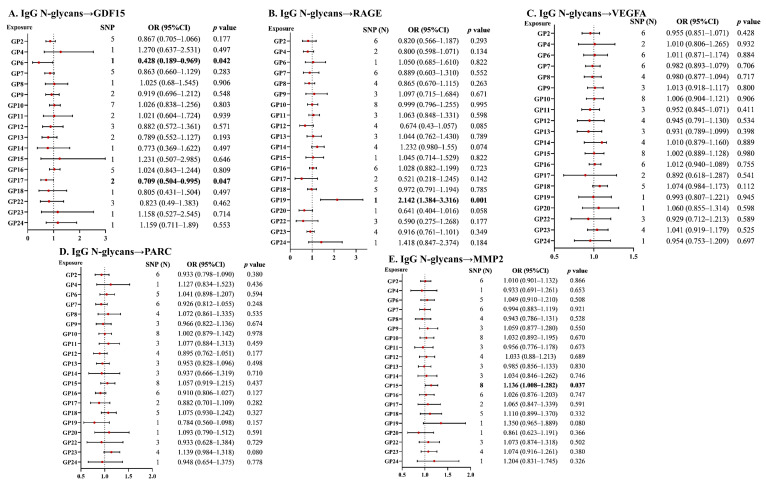
The causal associations of IgG N-glycans with SASP according to forward MR analyses. CI: confidence interval; GDF15: growth/differentiation factor 15; GP: glycan peak; IgG: immunoglobulin G; MMP2: matrix metalloproteinase 2; MR: Mendelian randomization; PARC: pulmonary and activation-regulated chemokine; RAGE: advanced glycosylation end-product-specific receptor; SNP: single nucleotide polymorphism; SASP: senescence-associated secretory phenotype; and VEGFA: vascular endothelial growth factor A.

Genetic liability to GP6 was significantly associated with GDF15 according to the Wald ratio method (OR = 0.428, 95% CI = 0.189–0.969; *p* = 0.042; Figure 1 and Table S5). We observed that genetically determined GP17 was associated with GDF15 using two independent SNPs (fixed-effects IVW model OR = 0.709, 95% CI = 0.504–0.995, *p* = 0.047; Figure 1 and Table S5). In sensitivity analyses, the causal associations identified using the MR-RAPS method were marginally significant (OR = 0.707, 95% CI  = 0.495–1.008, *p* = 0.056; Table S5). The MR analysis using the Wald ratio method revealed that GP19 was significantly associated with RAGE (OR = 2.142, 95% CI = 1.384–3.316, *p* = 0.001; Figure 1 and Table S7). Genetically predicted that GP15 was significantly associated with high levels of MMP2 using IVW methods (IVW fixed-model OR = 1.136, 95% CI = 1.008–1.282; *p* = 0.037; Figure 1), while it was inconsistent with the sensitivity analyses (Table S13). The IVW method provided no evidence for putative causal associations of IgG N-glycans on VEGFA and PARC (Figure 1), and the results of sensitivity analyses generated similar associations (Tables S9 and S11).

As for pleiotropy tests, no evidence of directional pleiotropy was observed by MR–Egger regressions or MR Pleiotropy RESidual Sum and Outlier test (MR-PRESSO) for associations of IgG N-glycans with GDF15, PARC, and MMP2 (Tables S6, S12 and S14, all *p* > 0.05). Although we found that SNPs might affect the overall MR estimate in the leave-one-out analyses of IgG N-glycans with the outcomes of GDF15, PARC, and MMP2 (Figures S1, S4 and S5), the results were not further confirmed by the MR–Egger and MR-PRESSO methods. However, leave-one-out analyses and the MR–Egger method revealed the potential pleiotropy of GP2 and GP7 with the outcome of RAGE (Figure S2 and Table S8). MR-PRESSO distortion test was further conducted to examine significant differences in causal associations before and after outlier corrections, and the results were still significant (Table S8). In the pleiotropy testing of IgG N-glycans with VEGFA, we observed outliers (Figure S3). The MR-PRESSO method yielded similar results, with the *p* values of the global test for MR-PRESSO were less than 0.05 for GP6, GP10, and GP15 (*p* = 0.019, *p* = 0.035, and *p* = 0.028, respectively; Table S10). Notably, the results showed no statistically significant difference before or after outlier correction.

### 2.2. Reverse Associations of SASP with IgG N-Glycans

**Figure 2:**
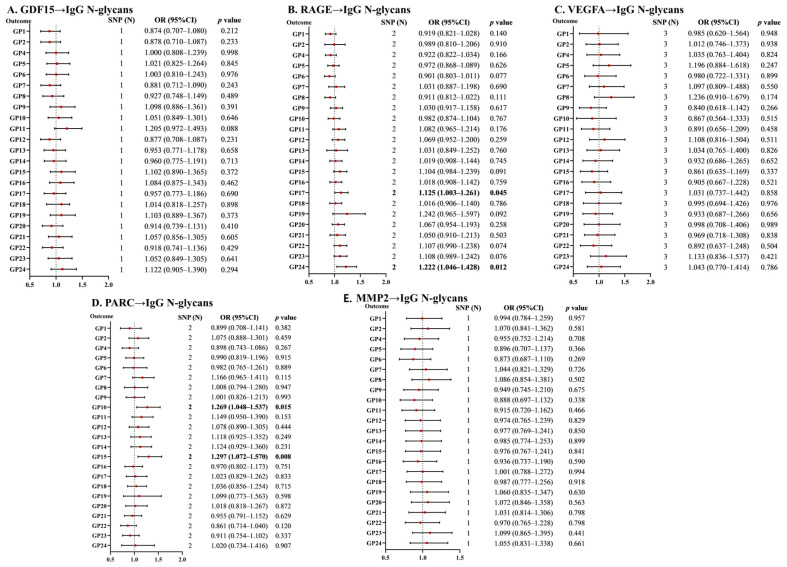
The causal associations of SASP with IgG N-glycans according to reverse MR analyses. CI: confidence interval; GDF15: growth/differentiation factor 15; GP: glycan peak; IgG: immunoglobulin G; MMP2: matrix metalloproteinase 2; MR: Mendelian randomization; PARC: pulmonary and activation-regulated chemokine; RAGE: advanced glycosylation end-product-specific receptor; SASP: senescence-associated secretory phenotype; SNP: single nucleotide polymorphism; and VEGFA: vascular endothelial growth factor A.

We performed MR analyses to evaluate the reverse causal relationships between IgG N-glycans and SASP. Only one GDF15-associated SNP was available and used to explore causal associations via the Wald ratio. As shown in Figure 2 and Table S15, no genetic causal association existed between GDF15 and IgG N-glycans. Similarly, the results revealed no associations between MMP2 and IgG N-glycans (Figure 2 and Table S20). For reverse MR analyses of VEGFA on IgG N-glycans, there were no causal associations reported (Figure 2 and Table S17). Genetically predicted RAGE was associated with GP17 and GP24 (OR = 1.125, 95% CI = 1.003–1.261, *p* = 0.045; OR = 1.222, 95%  CI  = 1.046–1.428, *p* = 0.012; Figure 2 and Table S16),which was consistent with the MR-RAPS method. The IVW analysis revealed significant causal associations between PARC and GP10 (OR = 1.269, 95% CI = 1.048–1.537, *p* = 0.015), and PARC on GP15 (OR = 1.297, 95%  CI = 1.072–1.57, *p* = 0.008; Figure 2 and Table S19). The analyses for the association of PARC performed by different methods yielded similar results to those by the method of IVW.

Although we observed potential pleiotropic effects of instrumental variables (Figure S6), there is no proof of the pleiotropy of VEGFA with IgG N-glycans in MR–Egger regressions for all *p* values > 0.05 (Table S18).

## 3. Discussion

This is the first MR study in which IgG N-glycosylation has been evaluated for causal associations with SASP. We used the framework of bidirectional MR to evaluate the causal relationships between IgG N-glycosylation and SASP. The forward MR analysis showed four pairs of IgG N-glycans and SASP associations (i.e., GP6→GDF15, GP17→GDF15, GP19→RAGE, and GP15→MMP2). We observed four pairs of SASPs on IgG glycans’ associations in reverse MR analyses (specifically, RAGE→GP17, RAGE→GP24, PARC→GP10, and PARC→GP15).

The present study identified causal roles between IgG N-glycome and SASP. In the previous study of 5117 people from four European groups, the degree of galactosylation showed the highest correlation with age [26]. The results showed that agalactosylated glycans increased with age but digalactosylated glycans declined. The correlation between monogalactosylated glycans and age was complicated, with some glycans rising, while others decreased due to the position of galactose and the presence of bisecting GlcNAc [26]. Despite noting the correlations between age and various elements of IgG N-glycosylation, the overall picture remains intricate and multifaceted. The relationships were broadly consistent with those in Europeans and populations of the Han Chinese [19]. It is noteworthy that the findings detected sex differences in the correlations of IgG N-glycan levels with age, and glycosylation varies more in females than in males. In addition, the aberrant glycosylation of IgG was observed significant associations with age-related diseases, including dementia, hypertension, and T2DM, while the characterization of IgG glycosylation showed increased levels of agalactosylated glycans and decreased levels of digalactosylated glycans [20]. Obviously, the mechanisms and regulation systems underlying IgG glycosylation in aging require further study.

IgG is a key effector protein of the human immune system and mediates pro- and anti-inflammatory activities through the engagement of its Fc fragment with distinct Fcg receptors (FcgRs) [27]. It was proven that elevated galactosylation may confer anti-inflammatory responses by binding to FcγRIIIb [10]. As for sialic acid, it was shown to be connected with a substrate of galactose deposits, and the sialylation of IgG maintained the anti-inflammatory activity [27]. Our findings showed completely consistent evidence in forward MR analyses that increased levels of galactosylation and sialylation (GP17) were significantly associated with decreased levels of GDF15, which played a vital part in cellular reaction to stress signals in cardiovascular diseases and presented positive associations with cardiovascular mortality [28]. The bisector GlcNAc, a branched-chain sugar residue in N-glycan, was displayed to have the effect of suppressing the biosynthesis of terminal residues, including fucose, sialic acid, and human killer-1 [29]. This might explain the positive associations of galactosylation and sialylation with bisecting GlcNAc (GP19) with RAGE, which has been shown to contribute to chronic diseases, like diabetes, amyloidoses, inflammatory conditions, and tumors, by advancing cellular dysfunction and binding to cellular surface receptors [30]. Considering that previous studies demonstrated that bisecting GlcNAc plays a part as a general suppressor of terminal modification, the positive association between galactosylation with bisecting GlcNAc (GP15) and MMP2 was expected.

In the reverse MR analyses, we found proof of causal associations between SASP and IgG N-glycans, implying that the aging process could impact the IgG N-glycome. Glycosylation is regulated by the relative levels of several biosynthetic (glycosyltransferases) and catabolic (glycosidases) enzymes [6]. β-Galactosidase (β-Gal), an enzyme that was used for the location of cellular senescence, can eliminate galactose [31]. The previous study revealed increasing β-Gal activity with advancing age in plasma samples from 230 healthy individuals aged between 55 and 87 years [32]. It has been hypothesized that increasing β-Gal activity during aging might lead to decreased galactosylation. However, contrary to expectations, the MR analyses did not provide supportive evidence. In addition, a study involving 13 healthy individuals spanning ages 25 to 86 years revealed no significant alterations in peripheral B cell galactosyltransferase activity with age [33]. Considering the availability of suitable samples for analysis and sample size, the evidence to support causal associations between changes in IgG N-glycosylation patterns and the activity of extracellular glycosyltransferases in the course of aging is still limited. The relationship between the activity of glycosyltransferases and IgG N-glycosylation patterns requires further investigation.

The present study reported bidirectional causal associations of IgG glycans and SASP, biomarkers of cellular senescence, suggesting potential regulatory mechanisms underlying IgG glycosylation and cellular senescence. However, our findings were still limited and need to be explored in further studies, including animal experiments and in population groups. In addition, our findings might provide practical utility for the identification of promising targets in anti-aging interventions. Exercise has been shown to cause changes in the IgG N-glycosylation profile, showing decreases in agalactosylated N-glycans, and an increase in dilactosylated and monosialylated N-glycans [34]. Further investigation should focus on the effect that IgG N-glycosylation is effect by anti-aging interventions. Moreover, the present study has notable strengths. The present study used SNPs as IVs to find causal evidence between the IgG N-glycome and SASP, which can provide causality in the condition of minimizing confounding bias and avoiding bias from reverse causation. Second, we used GWAS sources for exposure and outcomes with little overlap to guarantee the lowest type-1 error rate. We performed several sensitivity analyses to enhance the robustness of the IVW analysis in the bidirectional MR analyses. The limitations of this study must be declared. This study takes advantage of publicly available datasets from populations of European ancestry, and, therefore, the generalization of our finding is restricted in other ethnic groups. Second, it was shown that there were gender differences in observational studies in the associations between IgG N-glycans and aging, but gender-based genetic instruments are not available. Additionally, we obtained summary-level data between the IgG N-glycome and SASP, and therefore the results were analyzed with standard MR analytical methods that were based on linear assumptions. Researchers can take advantage of individual-level data of IgG N-glycans and SASP to clarify their nonlinear relationships. Unfortunately, other evidence, such as experimental data from populations to validate our conclusion, is scarce. Our findings should be considered as suggested evidence and need to be verified in a large cohort of individual.

## 4. Materials and Method

### 4.1. Bidirectional MR Design

**Figure 3:**
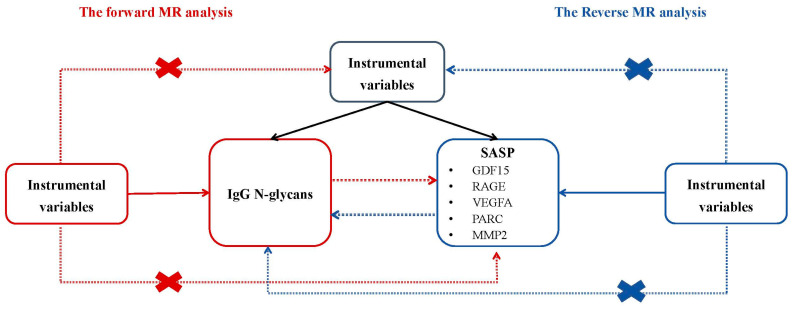
The overview of the bidirectional MR study design. MR: Mendelian randomization.

We set out to identify IVs of IgG N-glycans and SASP to include and employ the framework of MR and to explore bidirectional causal associations (Figure 3). For causal associations from MR studies, there are three assumptions that must be met: (1) selected IVs, namely genetic variants, are strongly associated with exposures; (2) the selected genetic variants are not supposed to be associated with any known or unknown confounders; and (3) those selected genetic variants must influence the outcome only through the exposure and not through any causal pathway. In addition, we reported an MR study according to the Strengthening the Reporting of Observational Studies in Epidemiology Using Mendelian Randomization (STROBE-MR) guidelines [35], and have provided a checklist of its items in Table S1.

### 4.2. Data Sources in Bidirectional MR Design

The GWAS data sources used in the bidirectional MR study of IgG N-glycans and SASP were drawn from the open publicly available GWAS datasets on people of European ancestry. Ethical approval was obtained from the corresponding original articles, as shown in Table 1, and was not provided in the present study.

#### 4.2.1. GWAS Data for IgG N-Glycans

Data for the IgG N-glycans were obtained from the largest GWAS with 8090 participants [36], which included 23 direct glycan traits (GP1-2, GP4-GP24), and those detailed structures and description are shown in Table S2. The data for measured N-glycan traits can be downloaded via the link provided by the researchers (https://datashare.ed.ac.uk/download/DS_10283_3238.zip), which was accessed on 21 December 2023 to perform the MR analyses.

#### 4.2.2. GWAS Data for SASP

We selected five senescence biomarkers, growth/differentiation factor 15 (GDF15), advanced glycosylation end-product-specific receptor (RAGE), vascular endothelial growth factor A (VEGFA), pulmonary and activation-regulated chemokine (PARC), and matrix metalloproteinase 2 (MMP2), which are reported by Sauver et al., since those traits provided the most strong increased associations with death for humans [5]. The genetic variants for five senescence biomarkers were obtained from publicly available data from recent GWASs on people of European ancestry, and measurements of SASP had been previously described in the original articles. We utilized the GWAS summary data for GDF15 from the study of 3394 European individuals, which was conducted by Folkersen et al. [37]. Data for VEGFA were accessed from the SCALLOP Consortium with 11 cohorts containing 14,744 European participants [39]. The GWAS summary statistics for RAGE (3301 individuals), PARC (5368 individuals), and MMP2 (5368 individuals) were acquired from the GWAS catalog (https://www.ebi.ac.uk/gwas/, accessed on 1 June 2024) or the IEU Open GWAS project (https://gwas.mrcieu.ac.uk/), those traits were reported in previous studies. All the datasets of SASP were accessed on 21 December 2023. Table 1 listed the detailed information of the study corresponding to IgG N-glycans and SASP.

### 4.3. Instrumental Variables Selection

The MR design requires instrumental variables associated with the exposure of interest (i.e., IgG N-glycans or SASP); in this case, we selected genome-wide significant (*p* < 5 × 10^−8^) SNPs used to match the hypothesis. Then, we clumped those SNPs based on the linkage disequilibrium (LD) of 1000 genomes of European samples and kept SNPs with the lowest *p* value (r^2^ < 0.001) as IVs. If the selected IVs were associated with the outcomes, these would be removed. Complete information on the selected IVs used in the directional MR design is presented in Tables S3 and S4.

### 4.4. MR Analyses

The causal relationships between IgG N-glycome and SASP were described by using odds ratios (ORs) and their 95% confidence intervals (CIs). We adopted the inverse-variance weighted method (IVW) and Wald ratio method as the main causal estimates. The Wald ratio method was used in the condition of one exposure SNP available for analysis; otherwise, the IVW method was adopted [41]. The Cochran Q statistic was adopted to test the heterogeneity between IVs selected in the bidirectional MR study [42]. Random-effects IVW models were used in the existing condition of heterogeneity; otherwise, fixed-effects IVW models were used. Notably, the IVW method is based on the hypothesis that all core assumptions of MR are valid. Consequently, we performed examinations by using the MR–Egger method [43], the weighted median method [44], the penalized weighted median (PWM) method [44], the MR-adjusted profile score (RAPS) method [45], and the MR-PRESSSO method [46], from which we could estimate causal relationships on the bias of different assumptions and test the robustness of the main methods. We employed the MR-PRESSSO and MR–Egger methods to test directional pleiotropy [43,46]. If directional pleiotropy existed, the intercept tested in the MR–Egger method differed from zero, and the *p* value was <0.05. Furthermore, the MR-PRESSO method was used to detect outlier SNPs and examine differences before and after outlier correction. In addition, we performed leave-one-out analyses to assess the potential influence of a particular variant on the causal associations between IgG N-glycans and SASP.

**Figure 4:**
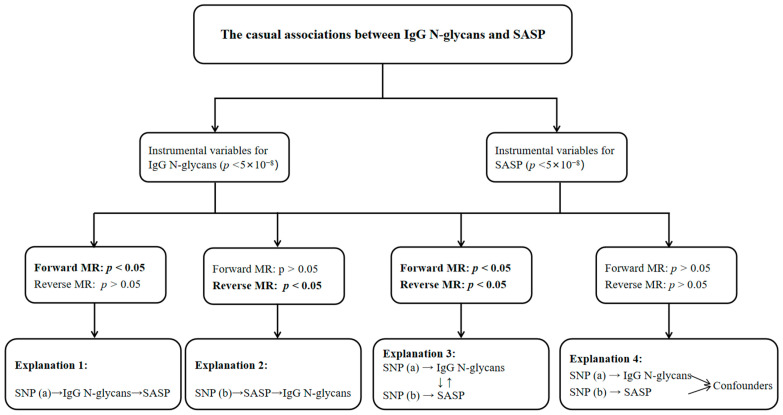
Analyses pipeline to evaluate the explanations for the causal associations between IgG N-glycans and SASP. MR: Mendelian randomization; SASP: senescence-associated secretory phenotype.

Figure 4 showed four potential explanations of the causal relationships expected in forward and reverse MR analyses. Explanation 1: the significant associations of genetically predicted IgG N-glycans on SASP. Explanation 2: the reverse causal associations between IgG N-glycans and SASP. Explanation 3: the bidirectional causality between IgG N-glycans and SASP (all *p* < 0.05). Explanation 4: no causal association in MR analyses (all *p* > 0.05). The MR analyses were based on using the “TwoSampleMR” package and R software (version 4.1.2).

## 5. Conclusions

In conclusion, our MR study provided suggested evidence between the IgG N-glycome and SASP, implying potential regulatory mechanisms underlying IgG glycosylation and cellular senescence. These findings suggest that the IgG N-glycome is a potential target for anti-aging interventions to delay age-related diseases by regulating anti-inflammatory activity.

## Figures and Tables

**Table 1:** Basic information of summary statistics data sources in the bidirectional MR analyses.

Phenotype	No. of Participants	SNP (N)	Author	Year of Publication	PMID
IgG N-glycans	8090	2,574,846	Klarić et al. [36]	2020	32128391
GDF15	3394	5,270,646	Folkersen et al. [37]	2017	28369058
RAGE	3301	10,534,735	Sun et al. [38]	2018	29875488
VEGFA	14,744	12,958,278	Zhao et al. [39]	2023	37563310
PARC	5368	7,506,463	Gudjonsson et al. [40]	2022	35078996
MMP2	5368	7,506,463	Gudjonsson et al. [40]	2022	35078996

GDF15: growth/differentiation factor 15; MMP2: matrix metalloproteinase 2; MR: Mendelian randomization; PARC: pulmonary and activation-regulated chemokine; RAGE: advanced glycosylation end-product-specific receptor; SNP: single nucleotide polymorphism; and VEGFA: vascular endothelial growth factor A.

## Data Availability

All GWAS summary statistics used in this study are publicly available for download by qualified researchers, as shown in Table 1.

## Supplementary Materials

The following supporting information can be downloaded at https://www.mdpi.com/article/10.3390/ijms25126337/s1.
