# Supplementary material for: Bidirectional Two-Sample Mendelian Randomization Study of Immunoglobulin G N-Glycosylation and Senescence-Associated Secretory Phenotype
Source: Int J Mol Sci. 2024 Jun 7;25(12):6337. doi: 10.3390/ijms25126337 (PMC11203829; doi:10.3390/ijms25126337)
Supplement: Supplementary file 1 [file ijms-25-06337-s001.zip › Fig.legend.pdf]

Supplementary Materials:

Figure S1. MR leave-one-out analyses for IgG N-glycans on GDF15.

A: GP2; B: GP7; C: GP10; D: GP12; E: GP16; F: GP22. GDF15: growth/differentiation factor 15; GP: glycan peak.

Figure S2. MR leave-one-out analyses for IgG N-glycans on RAGE.

A: GP2; B: GP7; C: GP8; D: GP9; E: GP10; F: GP11; G: GP12; H: GP13; I: GP14; J: GP16; K: GP18; L: GP22; M: GP23. GP: glycan peak; RAGE: advanced glycosylation end product-specific receptor.

Figure S3. MR leave-one-out analyses for IgG N-glycans on VEGFA.

A: GP2; B: GP6; C: GP7; D: GP8; E: GP9; F: GP10; G: GP11; H: GP12; I: GP13; J: GP14; K: GP15; L: GP16; M: GP18; N: GP22; O: GP23. GP: glycan peak; VEGFA: vascular endothelial growth factor A.

Figure S4. MR leave-one-out analyses for IgG N-glycans on PARC.

A: GP2; B: GP6; C: for GP7; D: GP8; E: GP9; F: GP10; G: GP11; H: GP12; I: GP13; J: GP14; K: GP15; L: GP16; M: GP18; N: GP22; O:GP23. GP: glycan peak; PARC: pulmonary and activation-regulated chemokine.

Figure S5. MR leave-one-out analyses for IgG N-glycans on MMP2.

A: GP2; B: GP6; C: GP7; D: GP8; E: GP9; F: GP10; G: GP11; H: GP12; I: GP13; J: GP14; K: GP15; L: GP16; M: GP18; N: GP22; O: GP23. GP: glycan peak; MMP2: matrix metalloproteinase 2.

Figure S6. MR leave-one-out analyses for VEGFA on IgG N-glycans.

A: GP1; B: GP2; C: GP4; D: GP5; E: GP6; F: GP7; G: GP8; H: GP9; I: GP10; J: GP11; K: GP12; L: GP13; M: GP14; N: GP15; O: GP16; P: GP17; Q: GP18; R: GP19

S: GP20; T: GP21; U: GP22; V: GP23; W: GP24. GP: glycan peak; VEGFA: vascular endothelial growth factor A.
