# Supplementary material for: Bidirectional Two-Sample Mendelian Randomization Study of Immunoglobulin G N-Glycosylation and Senescence-Associated Secretory Phenotype
Source: Int J Mol Sci. 2024 Jun 7;25(12):6337. doi: 10.3390/ijms25126337 (PMC11203829; doi:10.3390/ijms25126337)
Supplement: Supplementary file 1 [file ijms-25-06337-s001.zip › Fig.S1-GDF15.pdf]

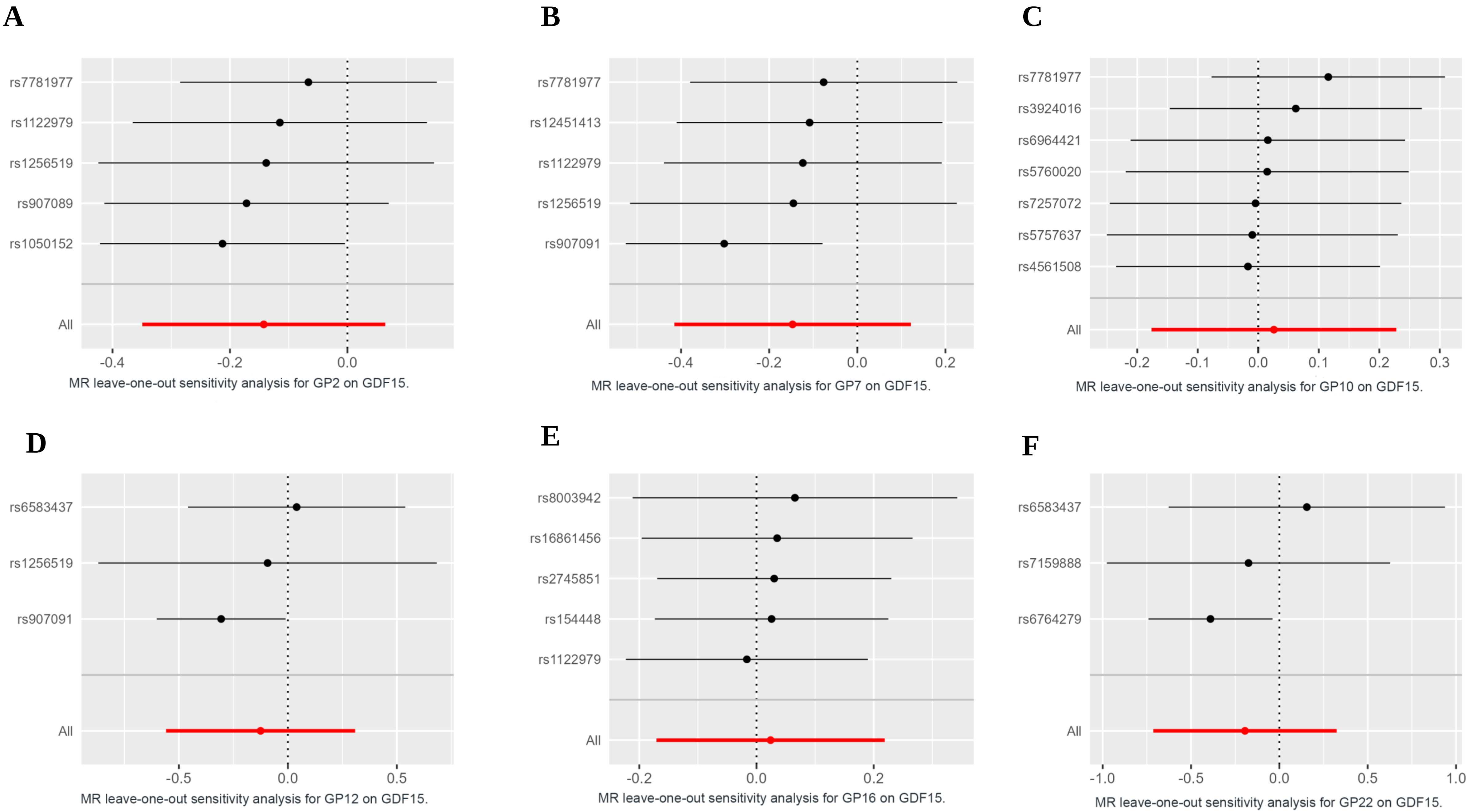
A
rs7781977
rs1122979
rs1256519
rs907089
rs1050152
All
-0.4
-0.2
0.0
MR leave-one-out sensitivity analysis for GP2 on GDF15.
B
rs7781977
rs12451413
rs1122979
rs1256519
rs907091
All
-0.4
-0.2
0.0
0.2
MR leave-one-out sensitivity analysis for GP7 on GDF15.
C
rs7781977
rs3924016
rs6964421
rs5760020
rs7257072
rs5757637
rs4561508
All
-0.2
-0.1
0.0
0.1
0.2
0.3
MR leave-one-out sensitivity analysis for GP10 on GDF15.
D
rs6583437
rs1256519
rs907091
All
-0.5
0.0
0.5
MR leave-one-out sensitivity analysis for GP12 on GDF15.
E
rs8003942
rs16861456
rs2745851
rs154448
rs1122979
All
-0.2
0.0
0.2
MR leave-one-out sensitivity analysis for GP16 on GDF15.
F
rs6583437
rs7159888
rs6764279
All
-1.0
-0.5
0.0
0.5
1.0
MR leave-one-out sensitivity analysis for GP22 on GDF15.
