# Supplementary figures and images for: Bidirectional Two-Sample Mendelian Randomization Study of Immunoglobulin G N-Glycosylation and Senescence-Associated Secretory Phenotype
Source: Int J Mol Sci. 2024 Jun 7;25(12):6337. doi: 10.3390/ijms25126337 (PMC11203829; doi:10.3390/ijms25126337)

**A**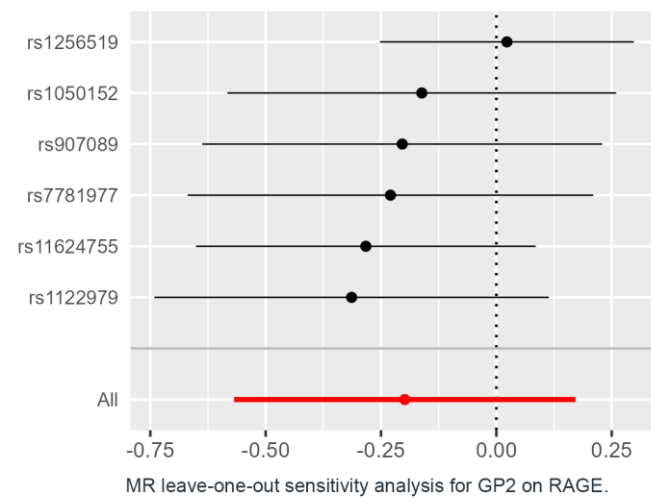**B**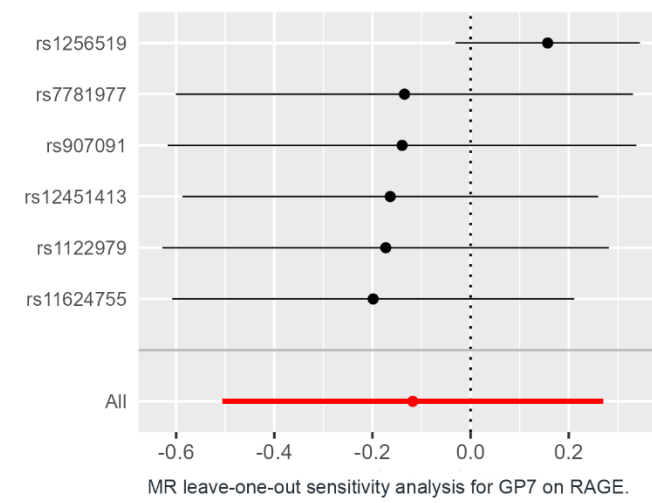**C**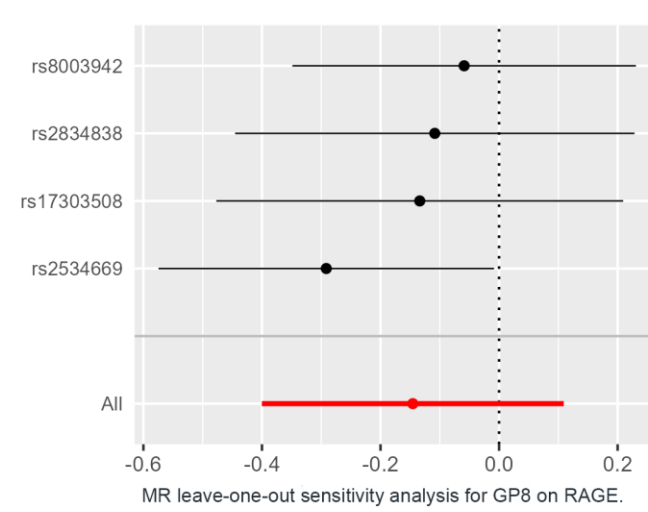**D**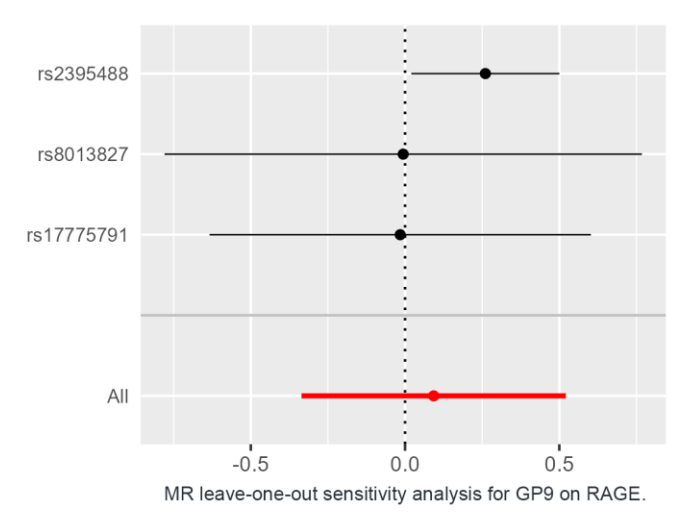**E**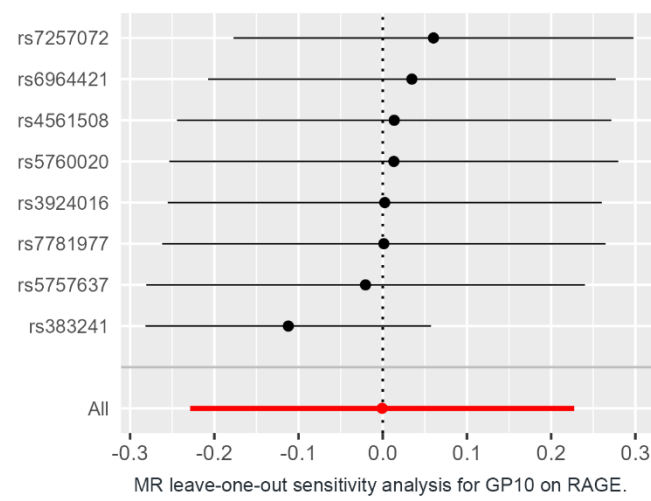**F**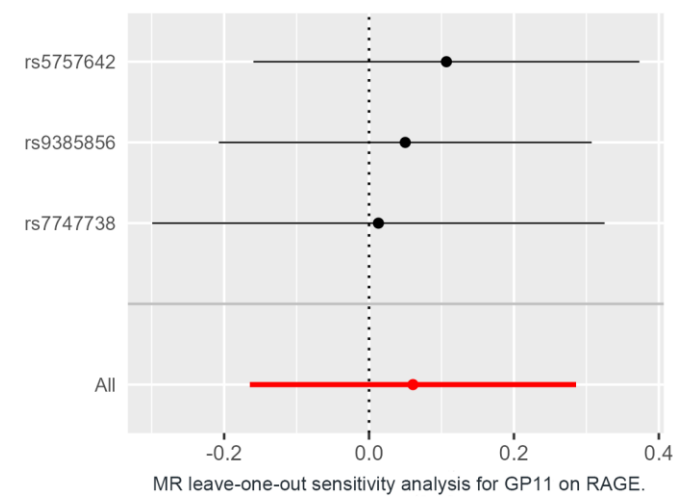**G**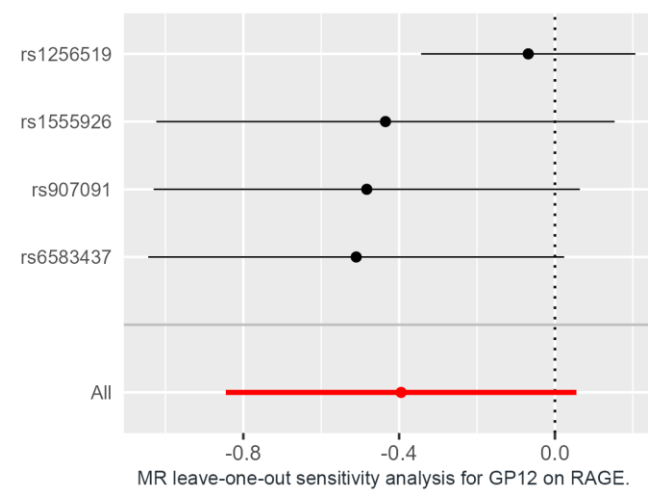**H**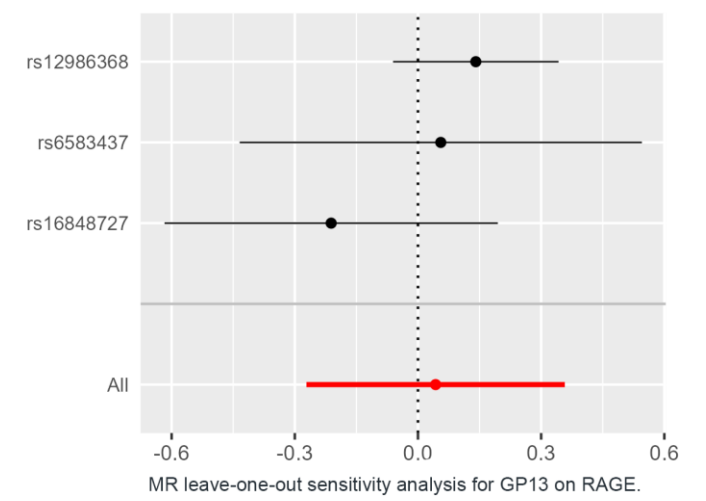**I**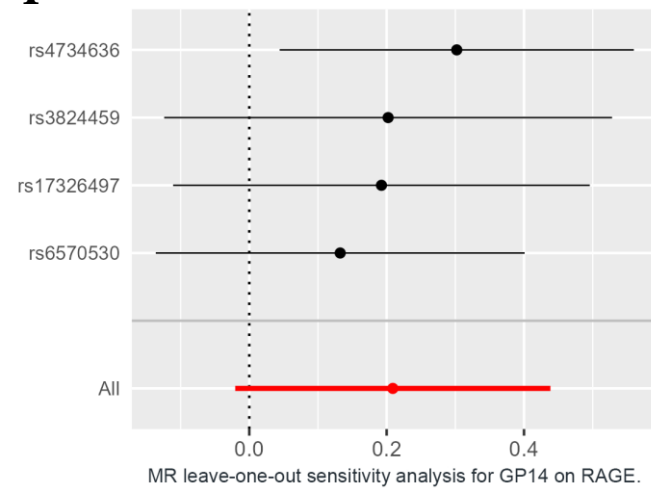**J**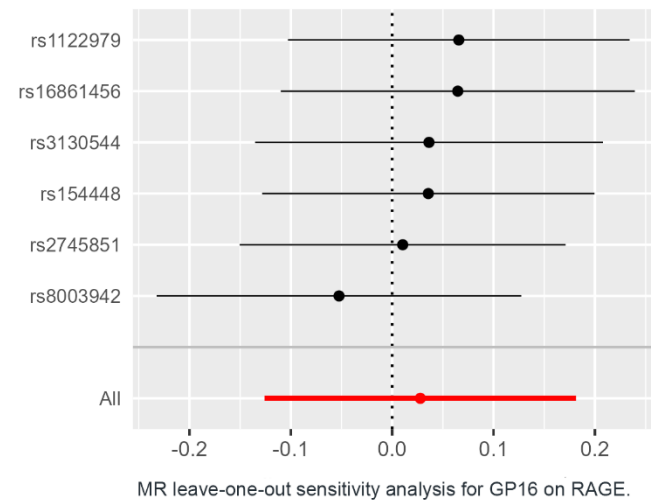**K**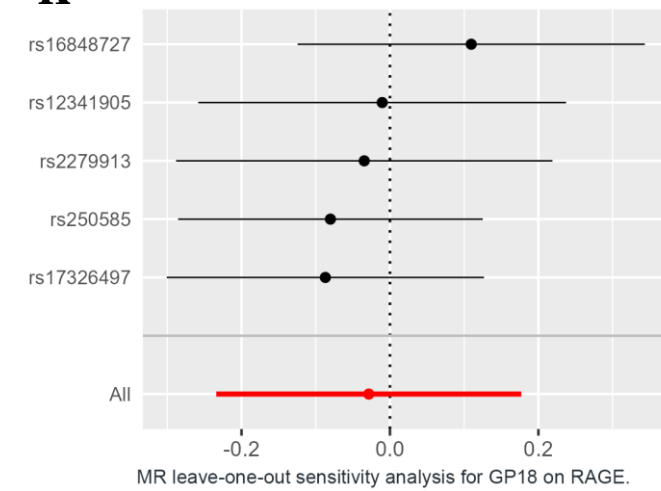**L**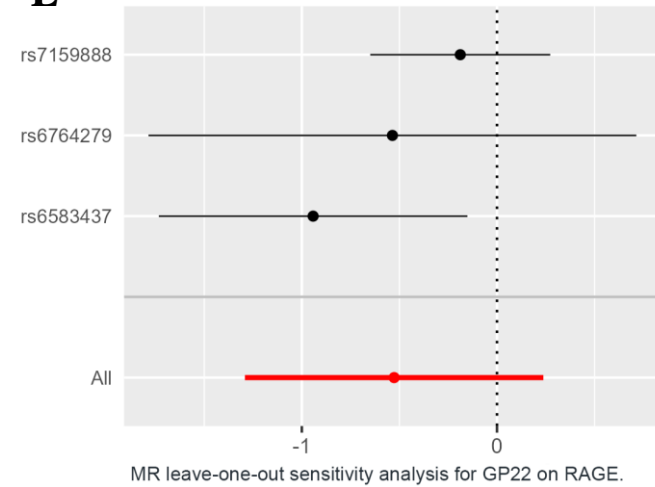**M**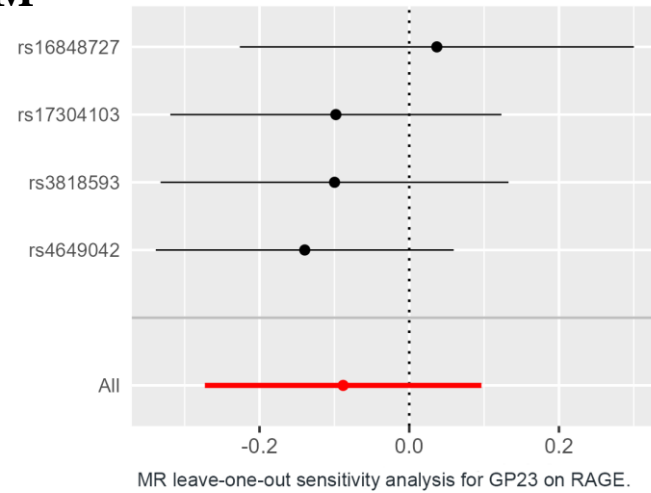

Supplement: Supplementary file 1 [file ijms-25-06337-s001.zip › Fig.S2-RAGE.pdf]

**A**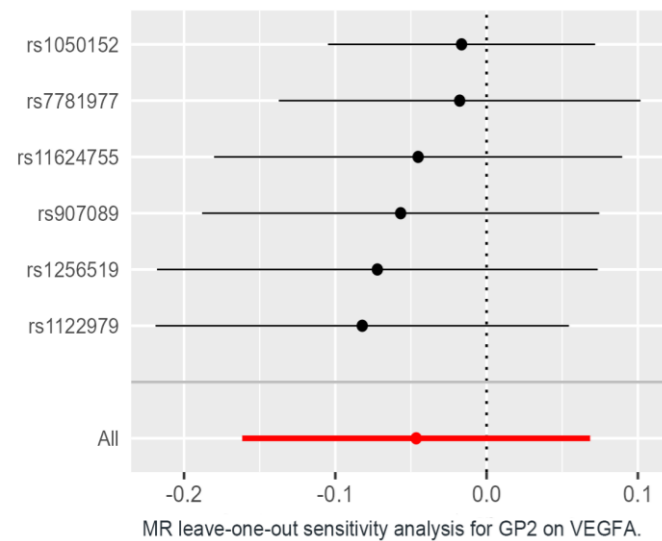**B**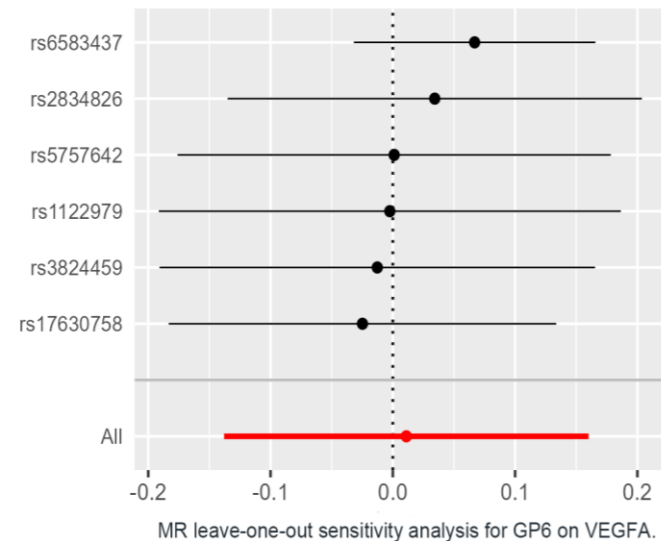**C**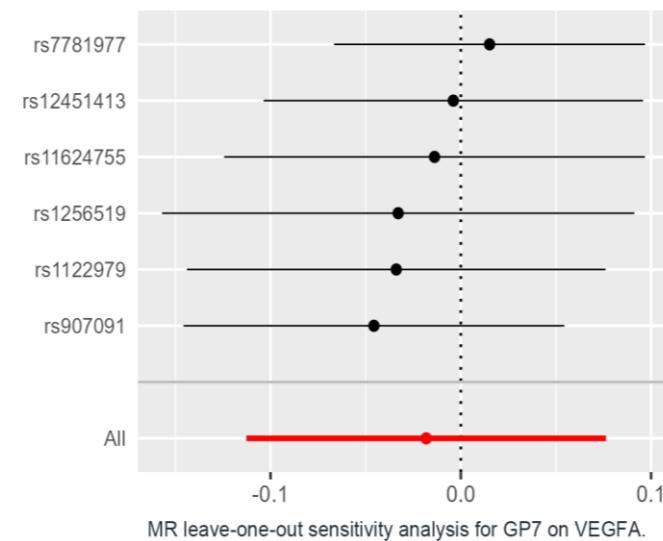**D**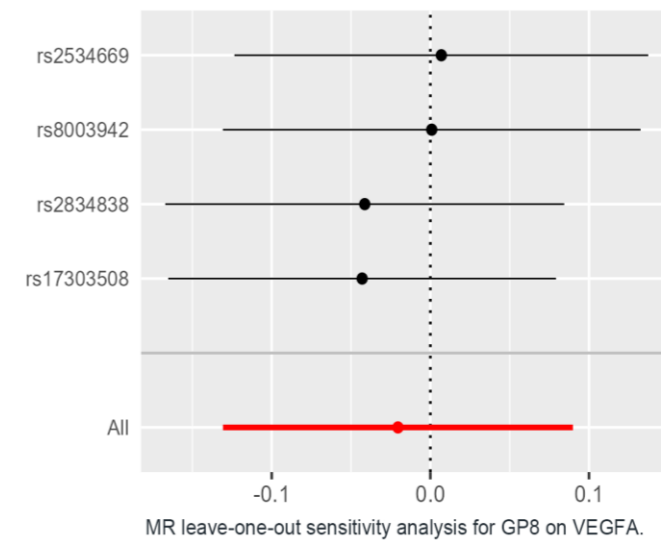**E**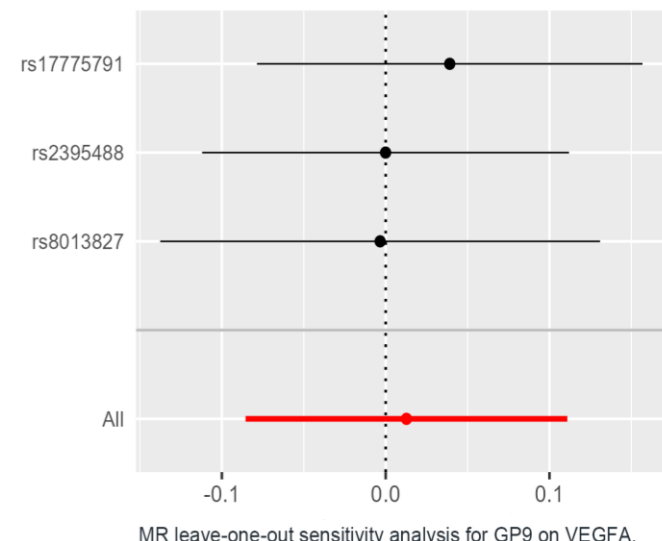**F**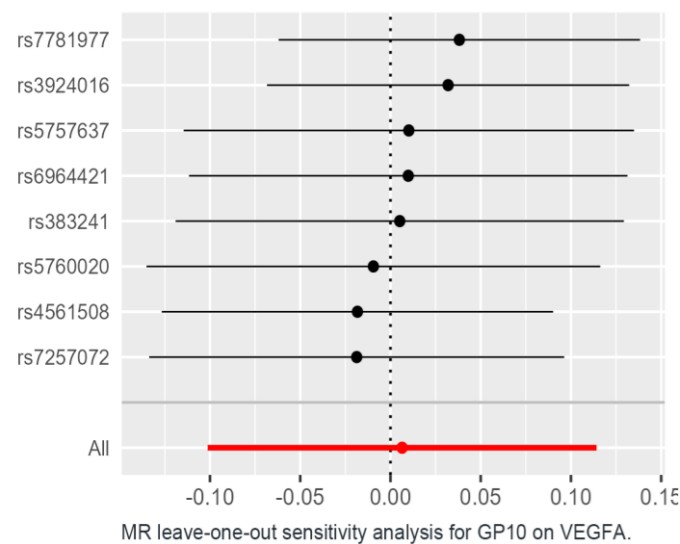**G**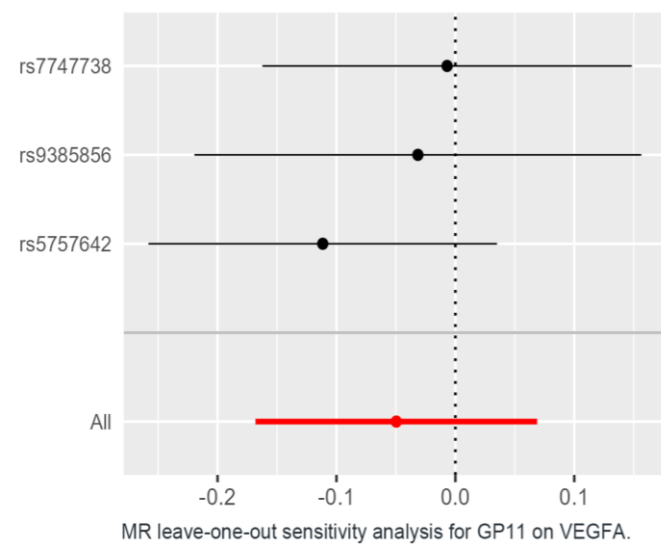**H**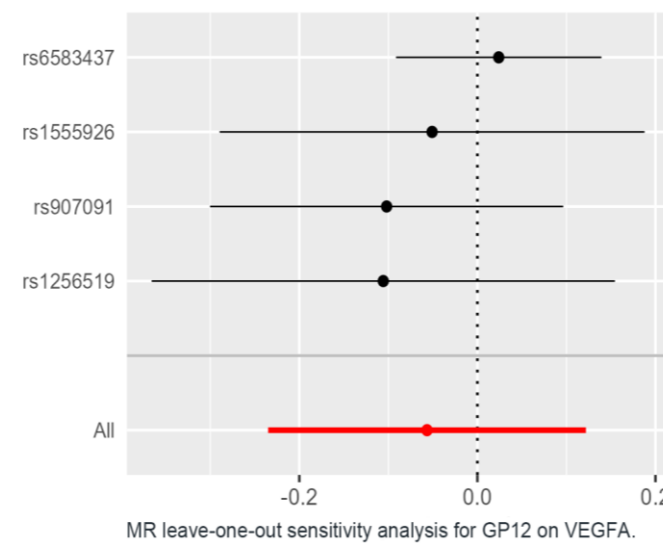**I**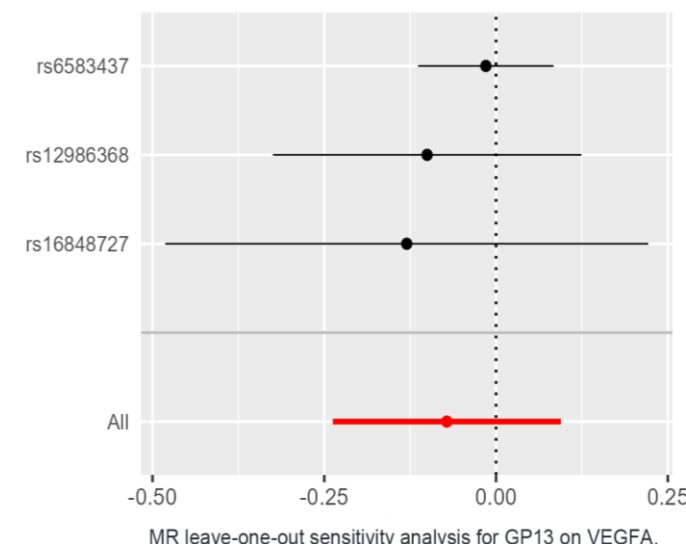**J**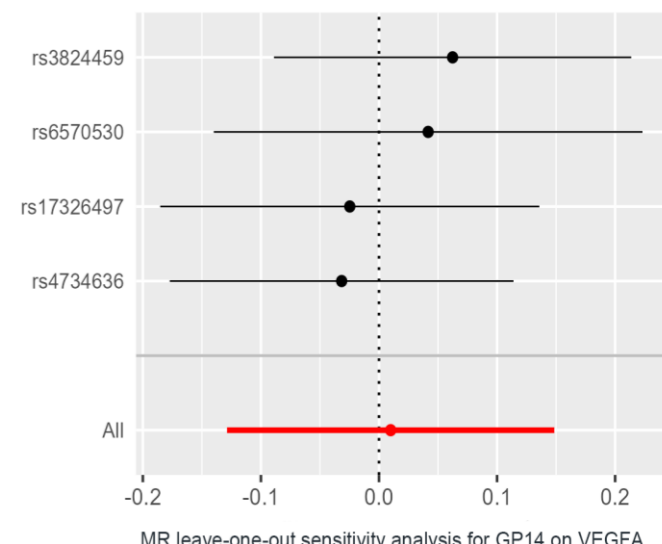**K**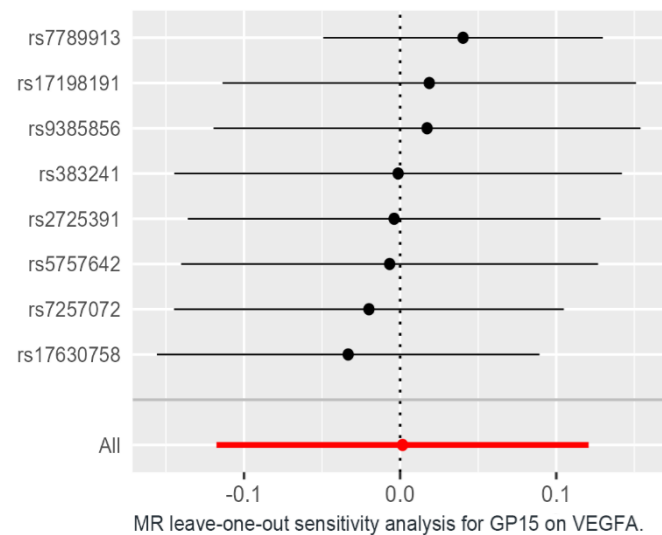**L**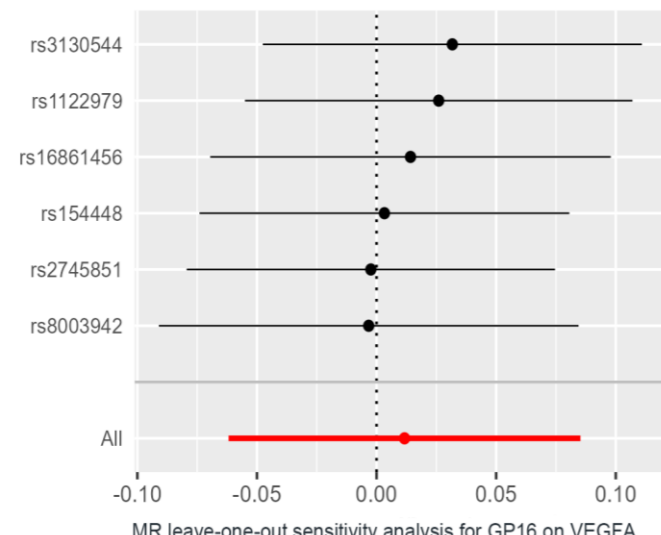**M**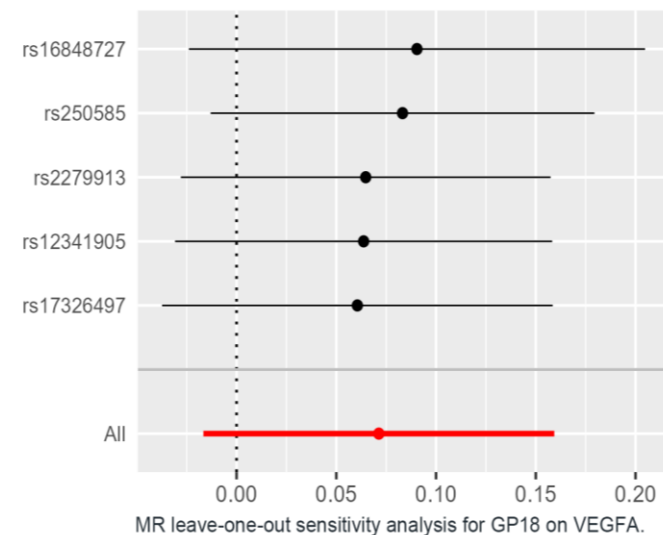**N**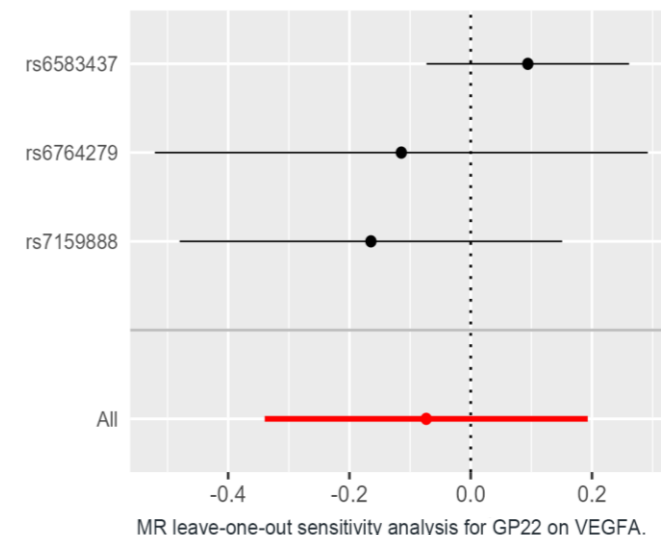**O**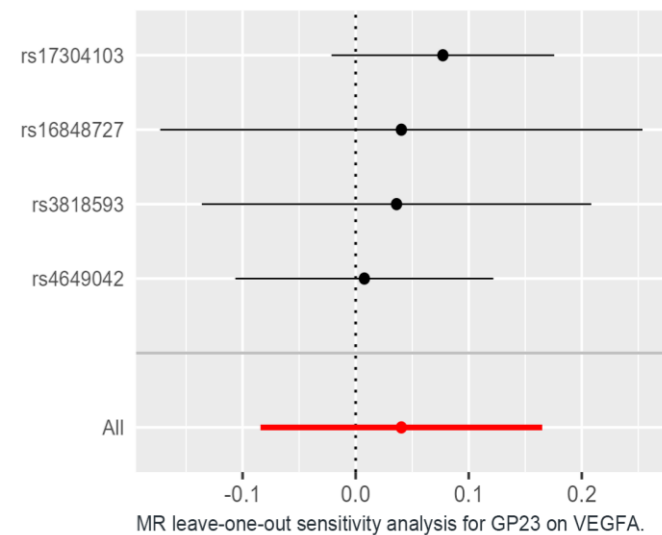

Supplement: Supplementary file 1 [file ijms-25-06337-s001.zip › Fig.S3-VEGFA.pdf]

**A**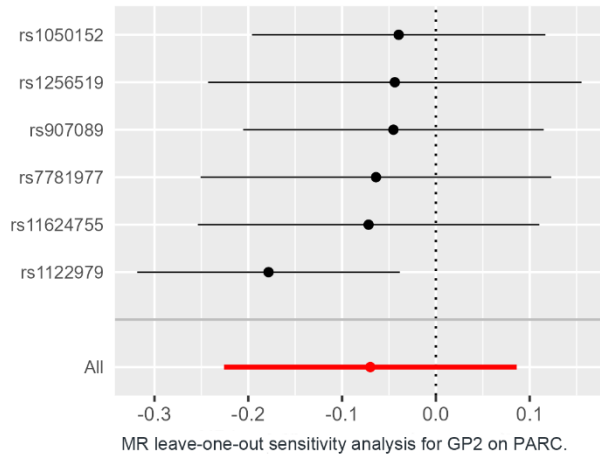**B**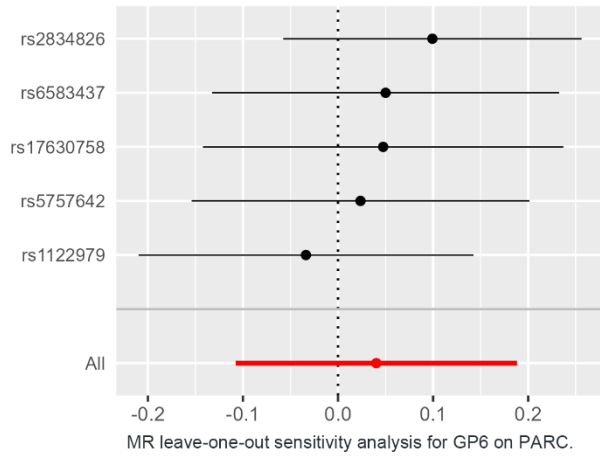**C**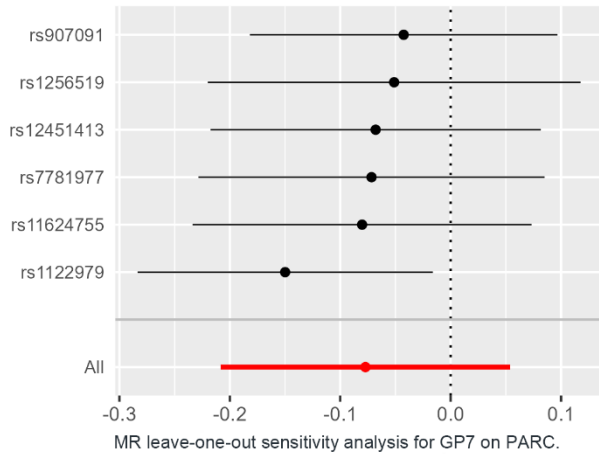**D**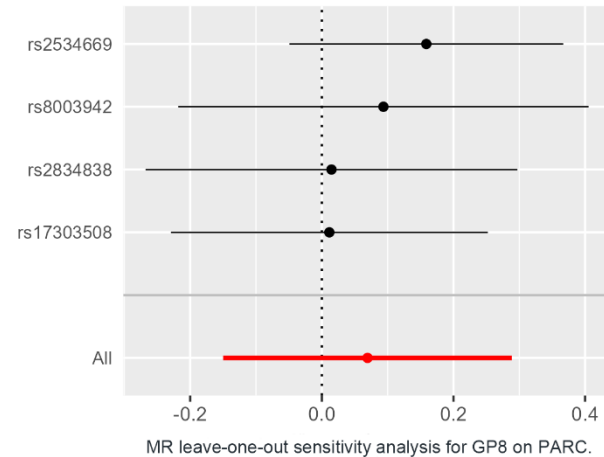**E**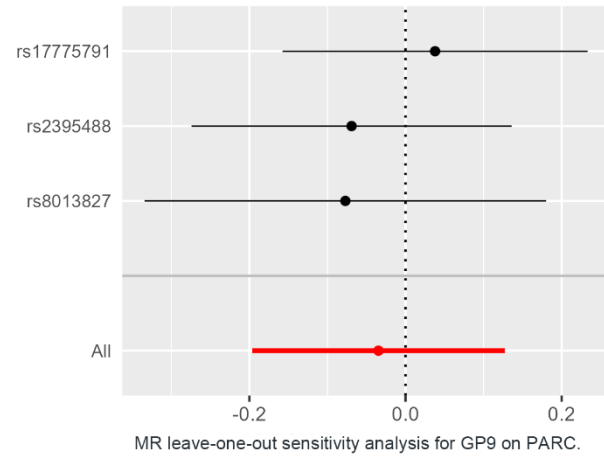**F**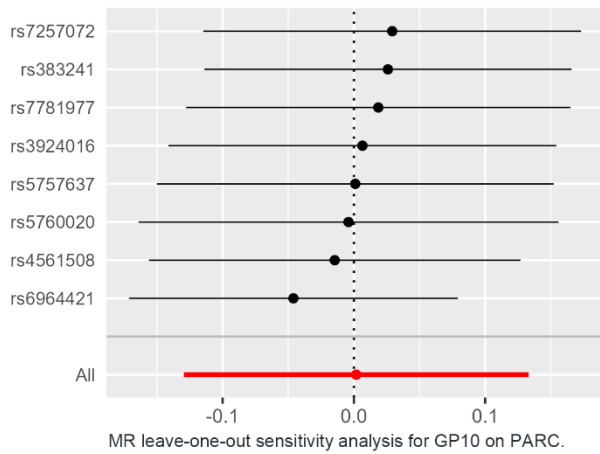**G**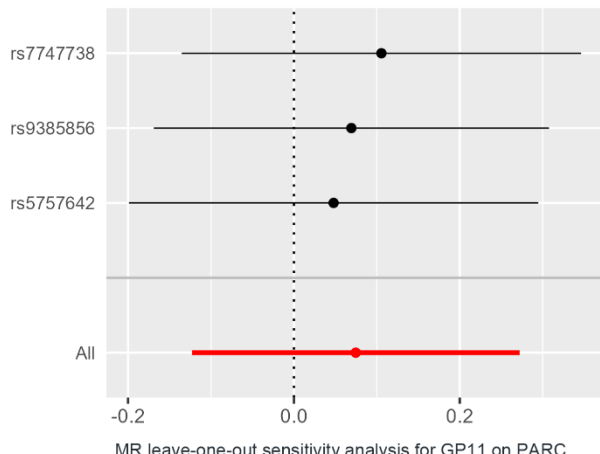**H**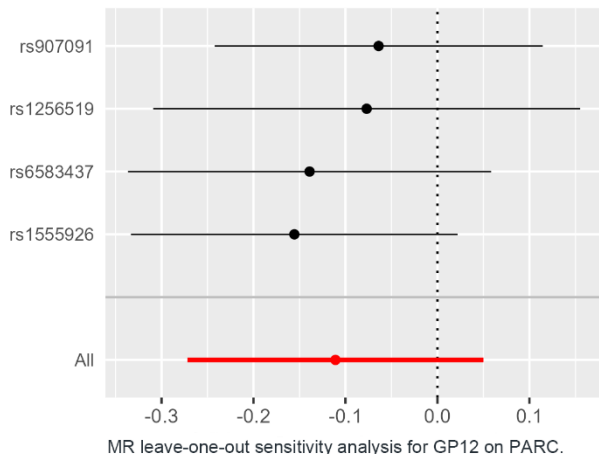**I**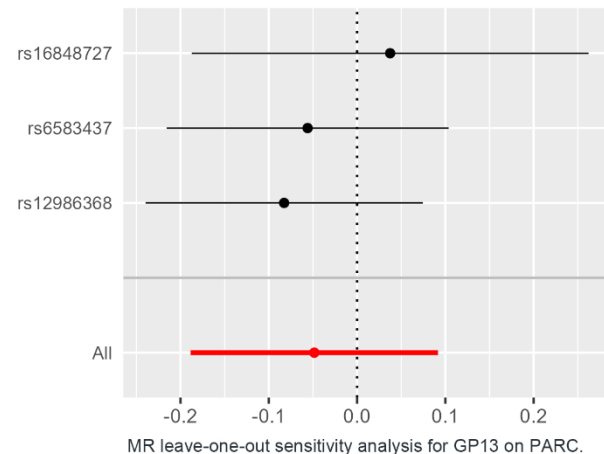**J**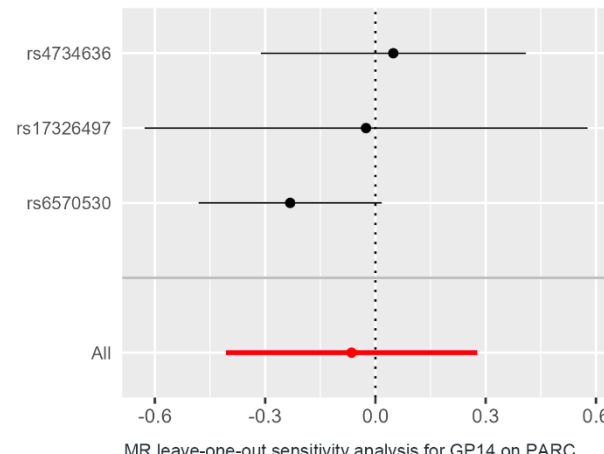**K**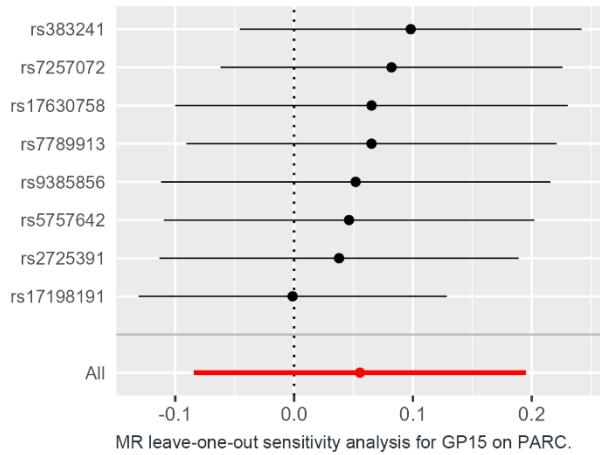**L**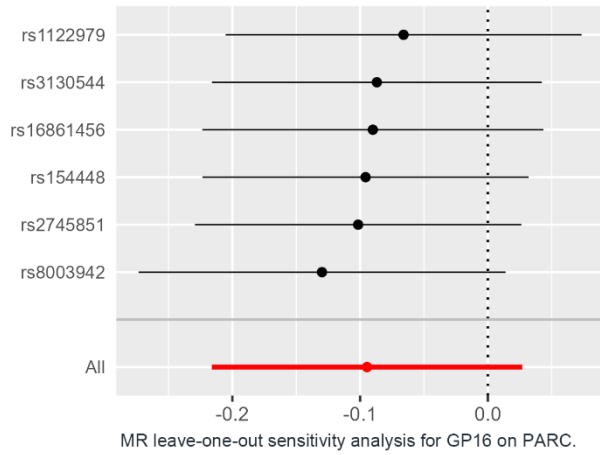**M**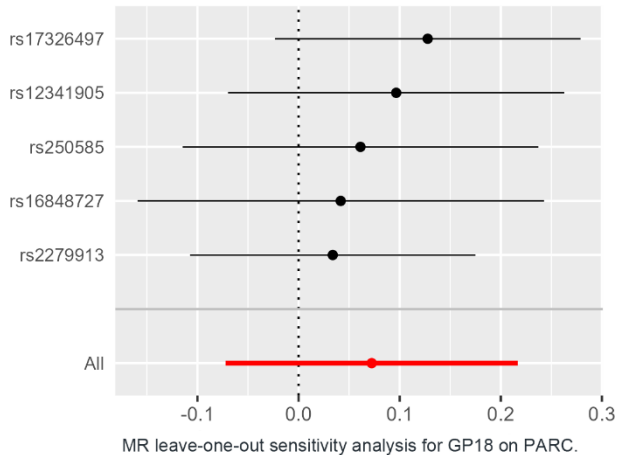**N**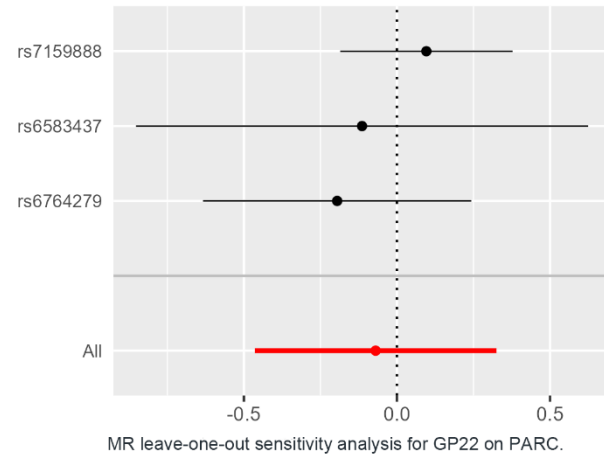**O**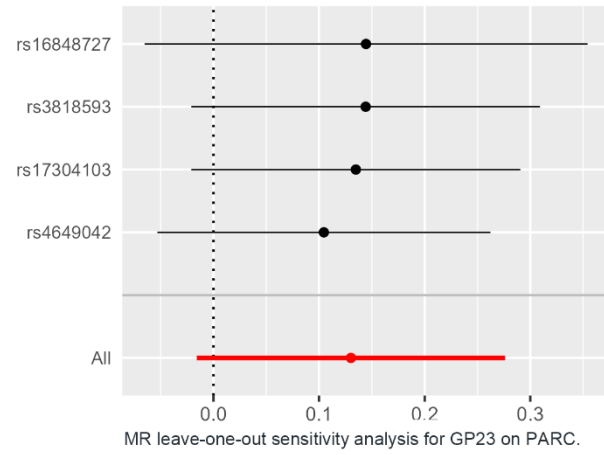

Supplement: Supplementary file 1 [file ijms-25-06337-s001.zip › Fig.S4-PARC.pdf]

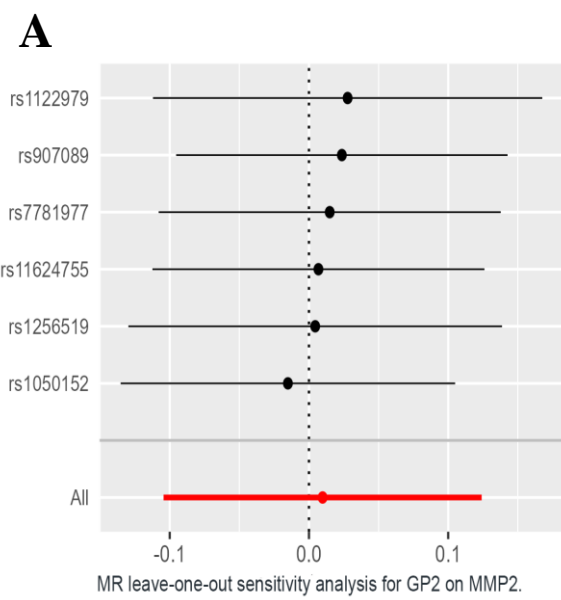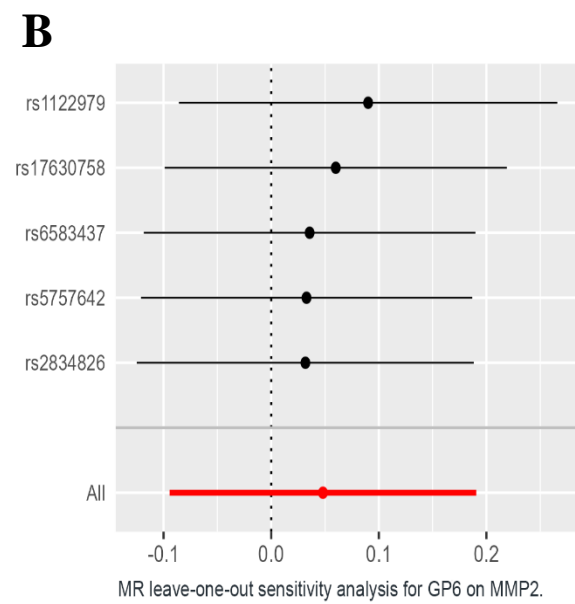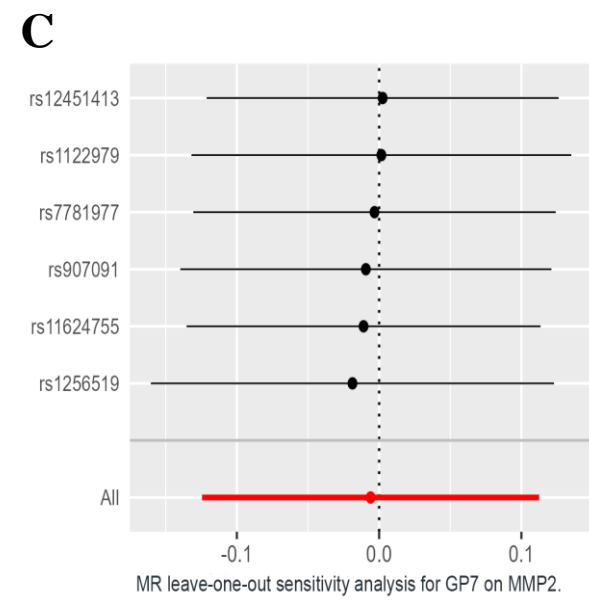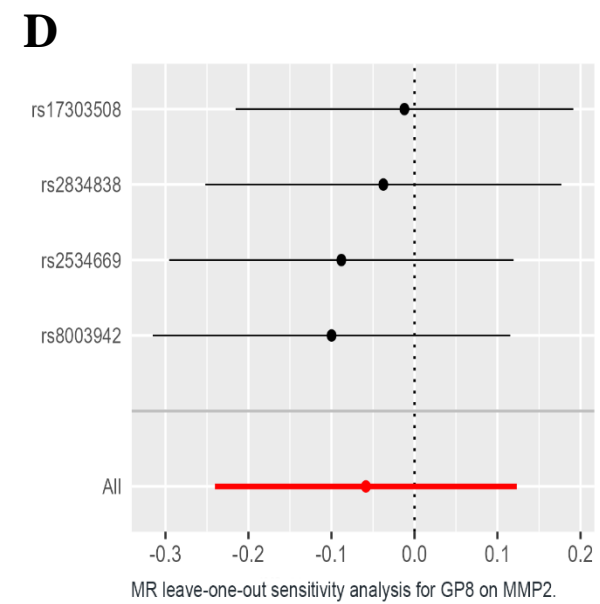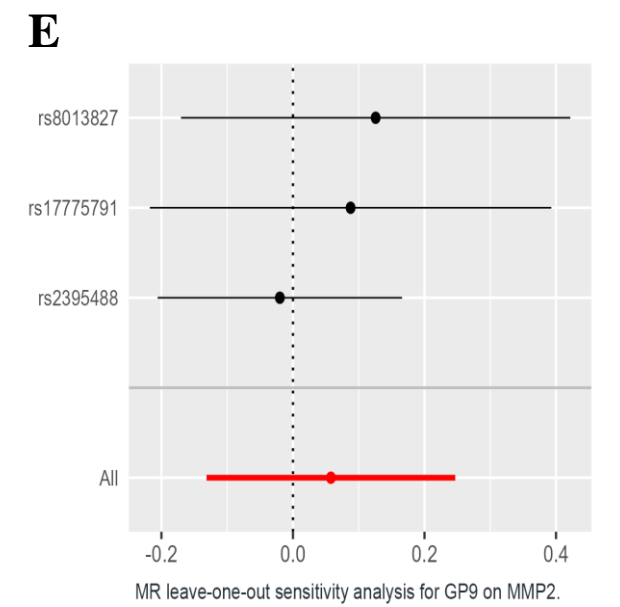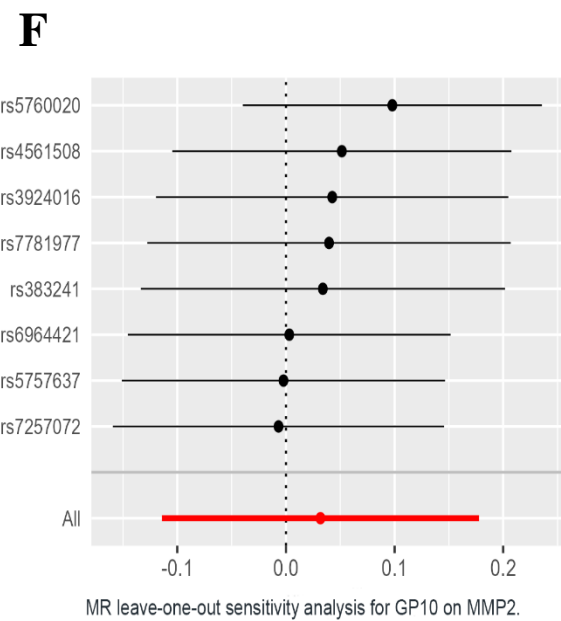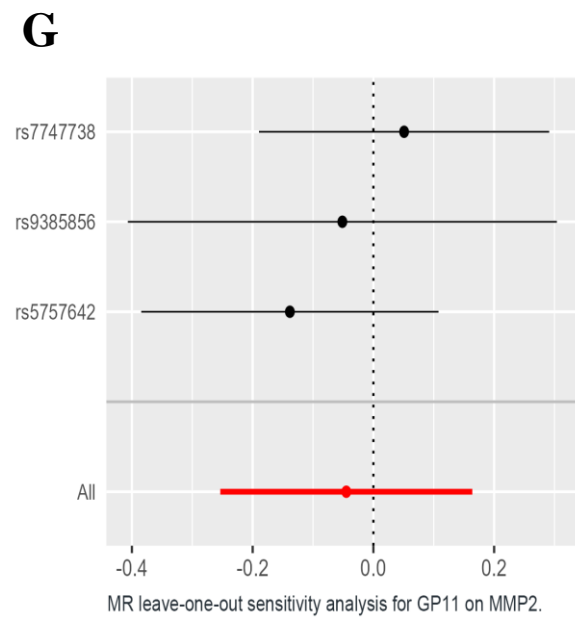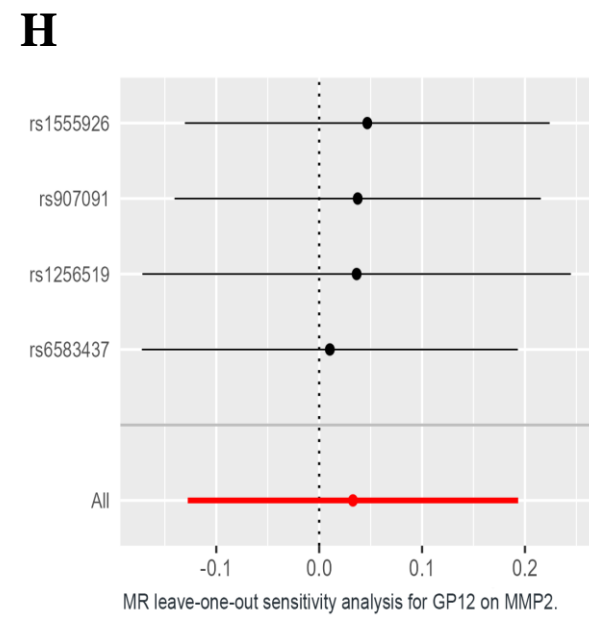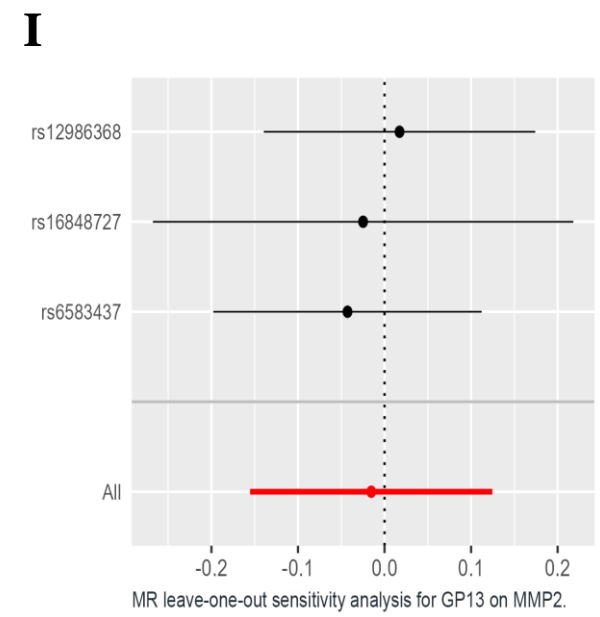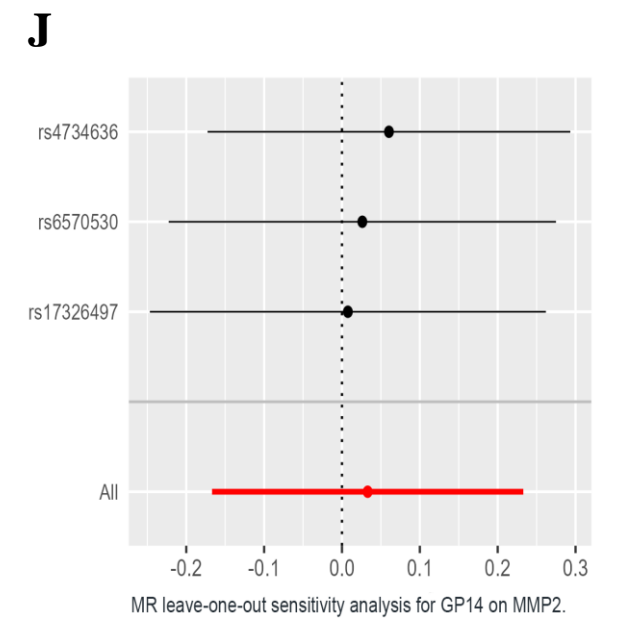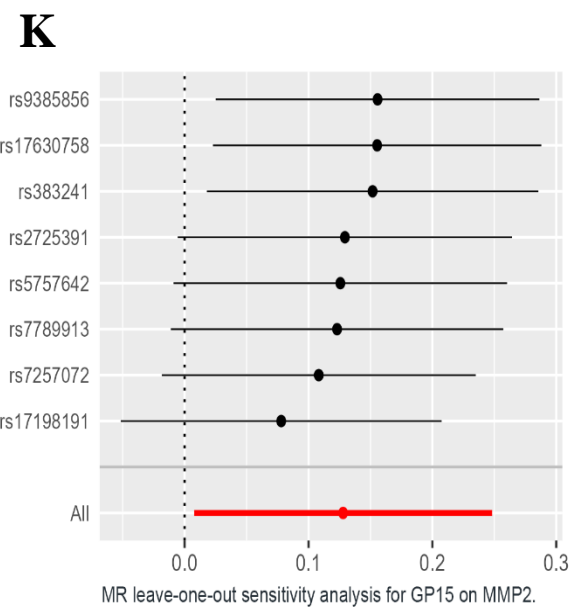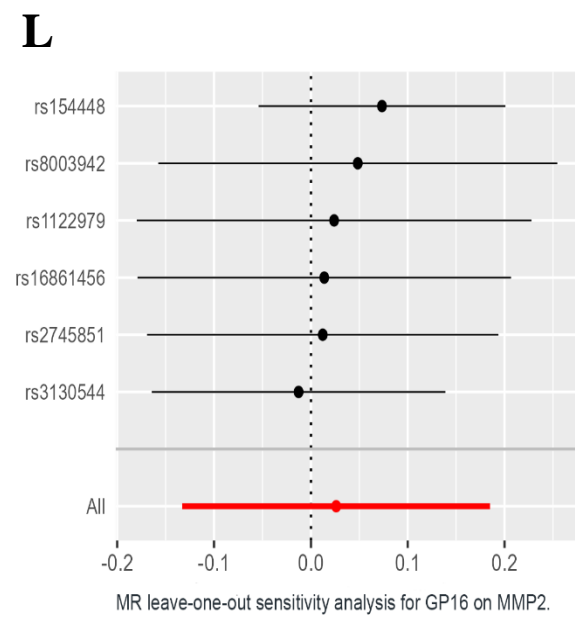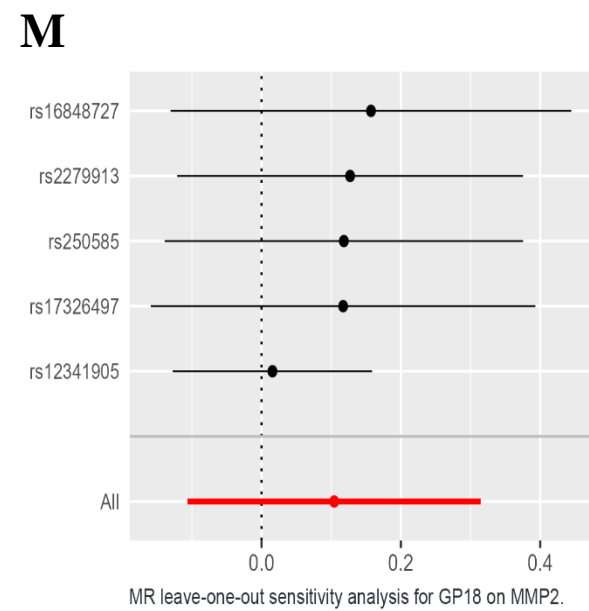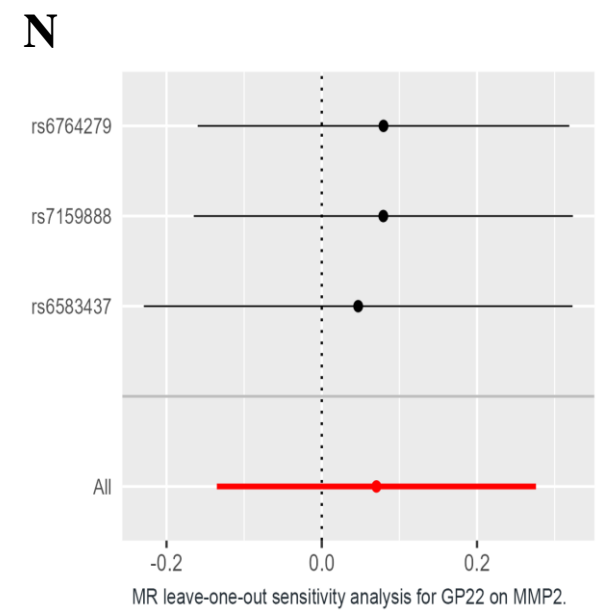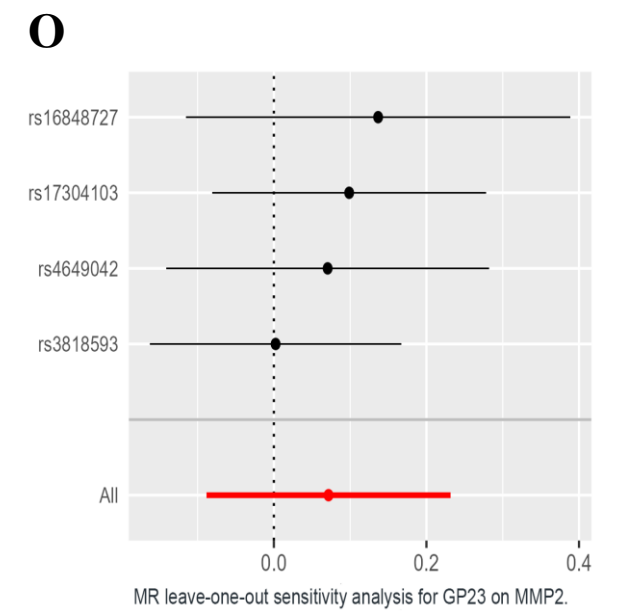

Supplement: Supplementary file 1 [file ijms-25-06337-s001.zip › Fig.S5-MMP2.pdf]

**A**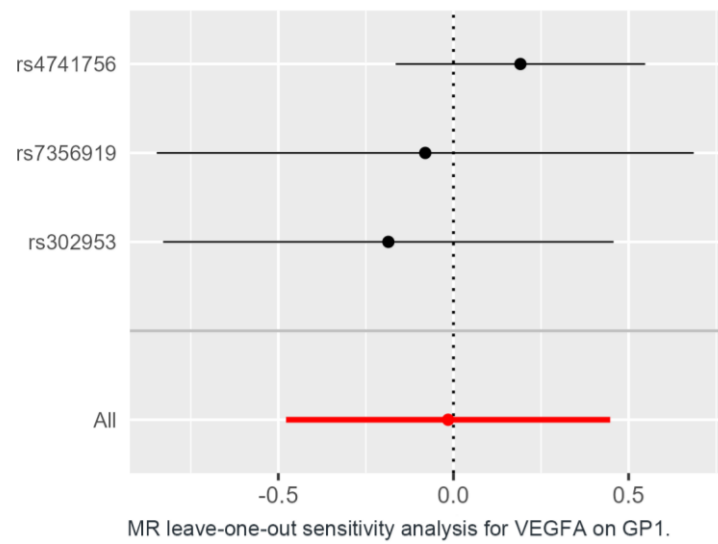**B**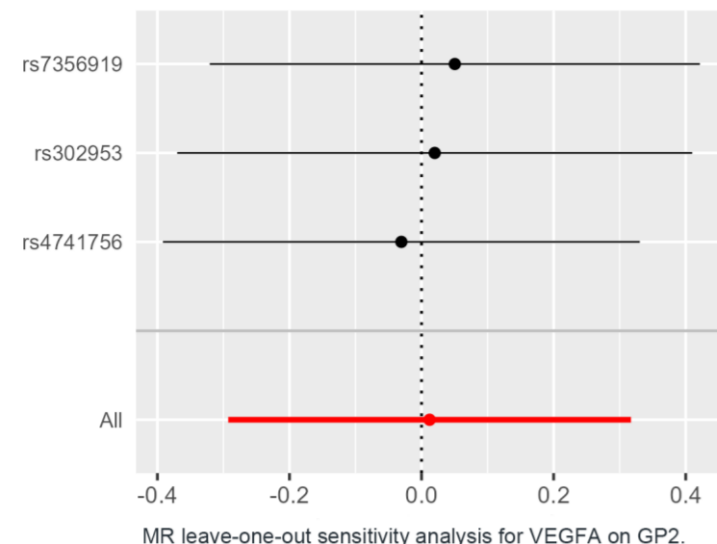**C**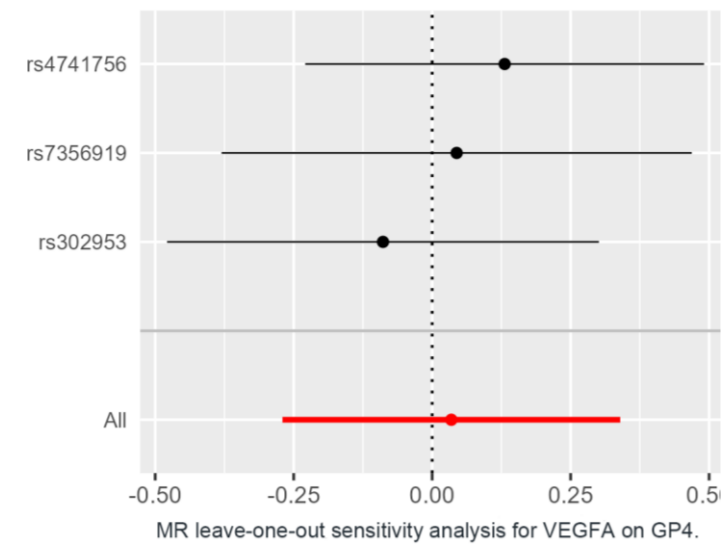**D**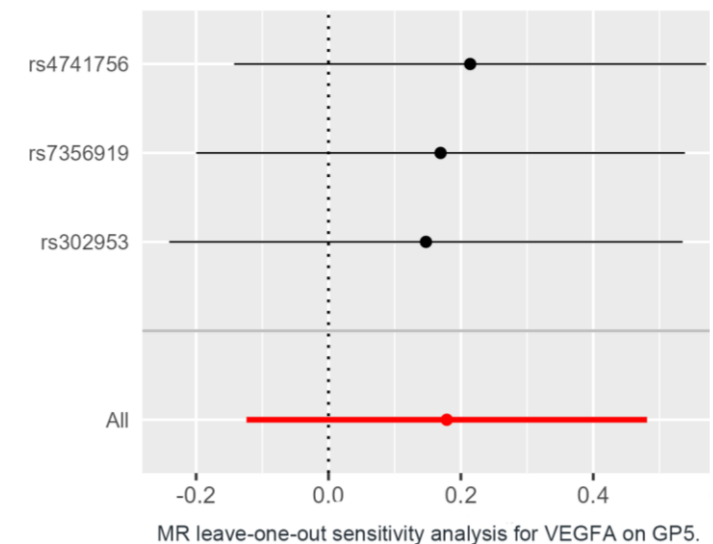**E**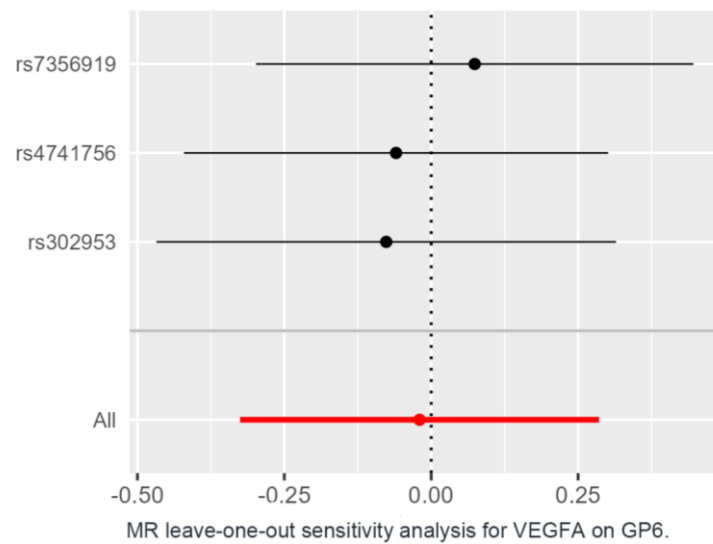**F**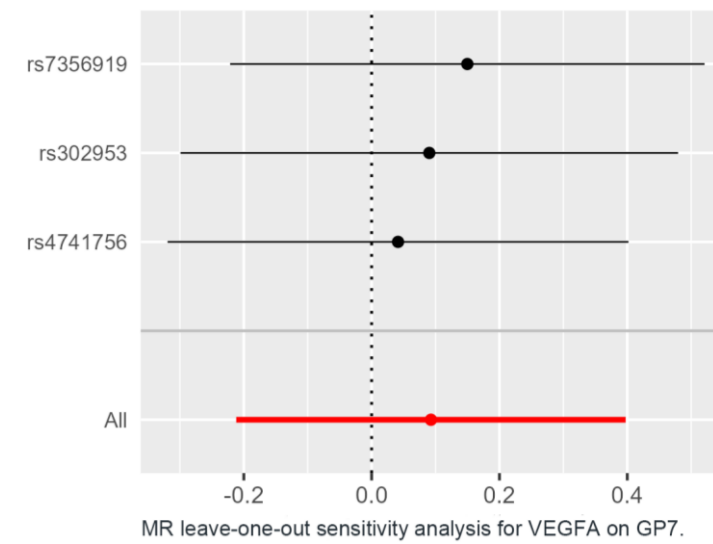**G**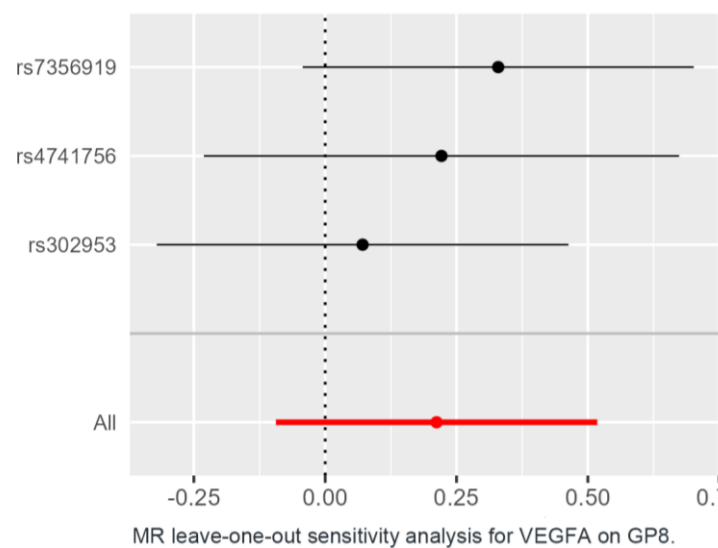**H**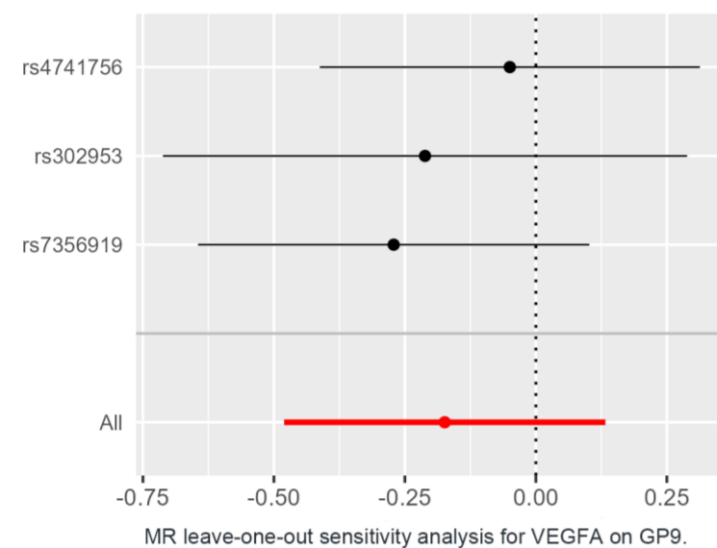**I**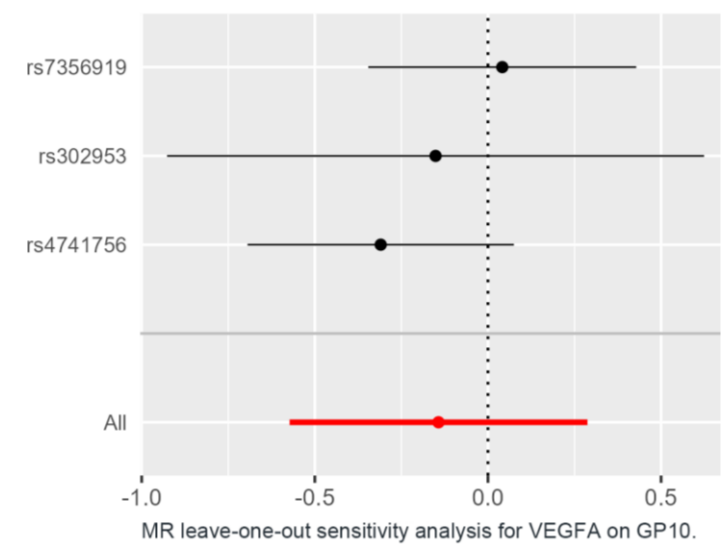**J**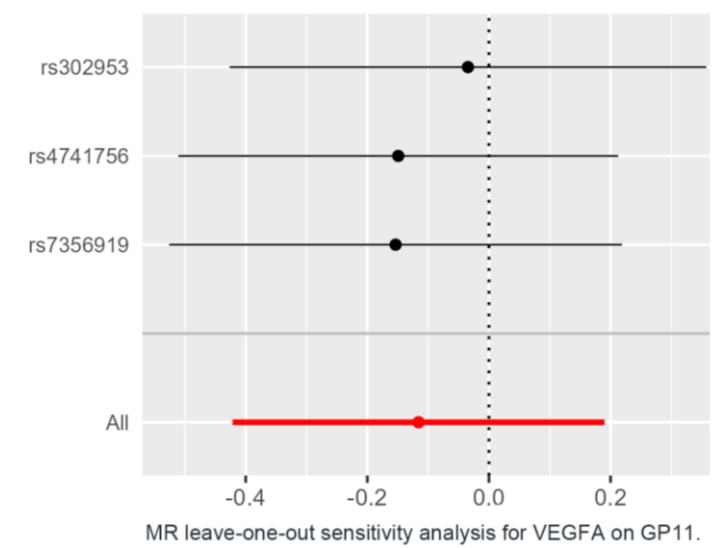**K**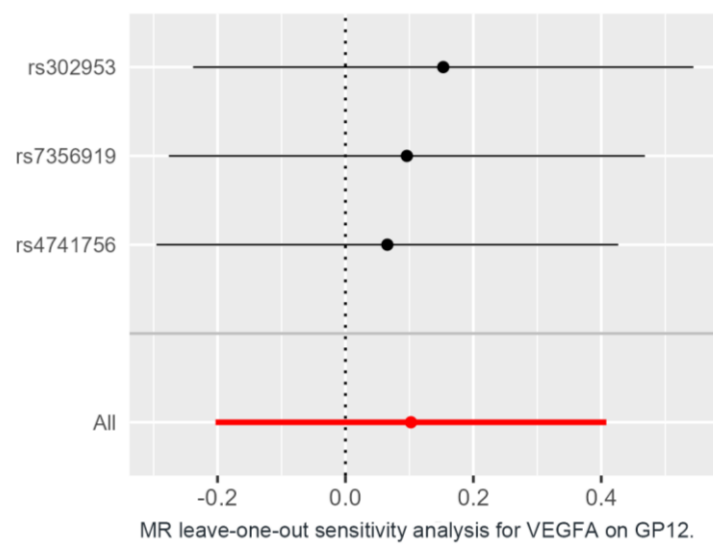**L**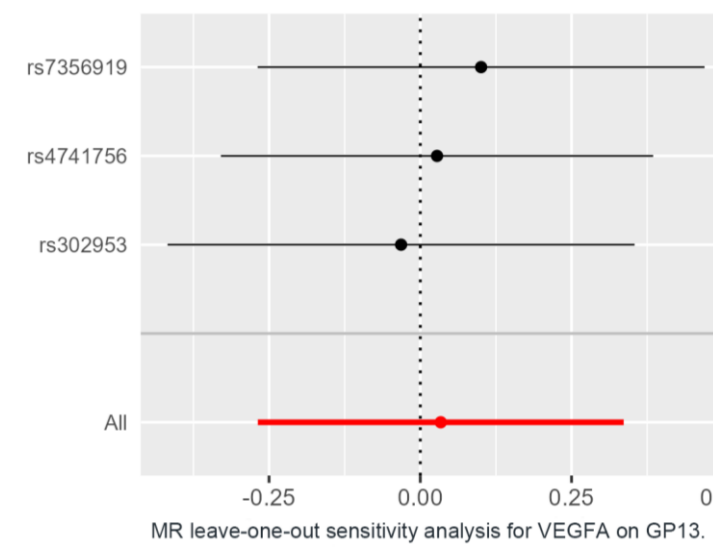**M**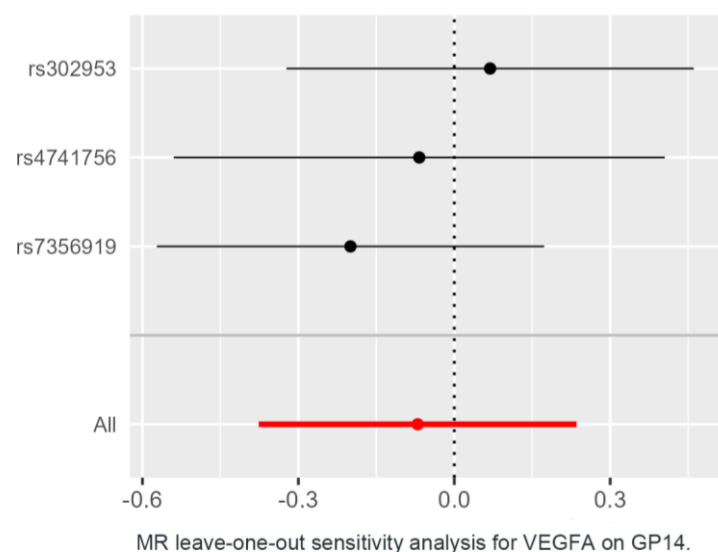**N**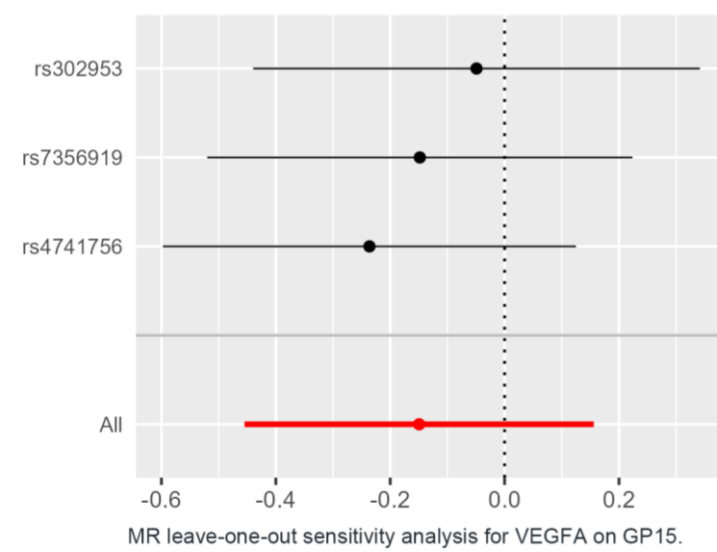**O**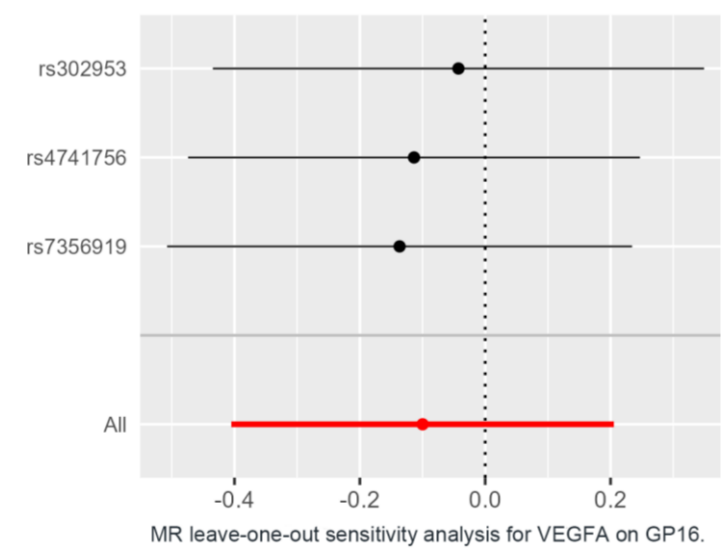**P**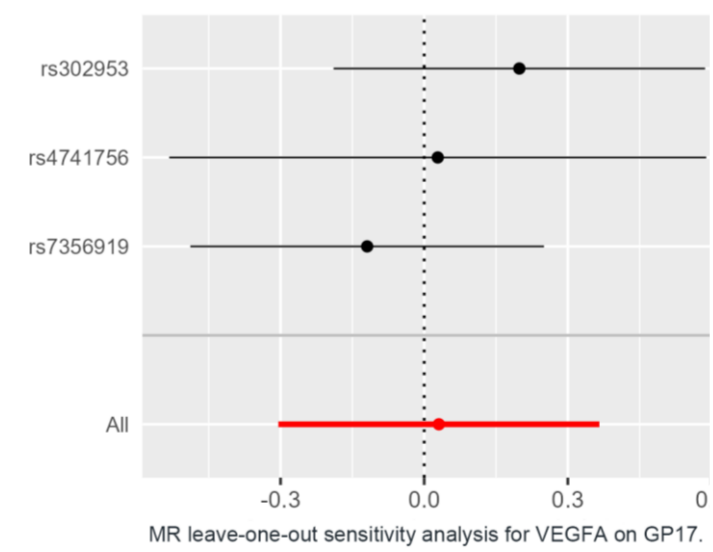**Q**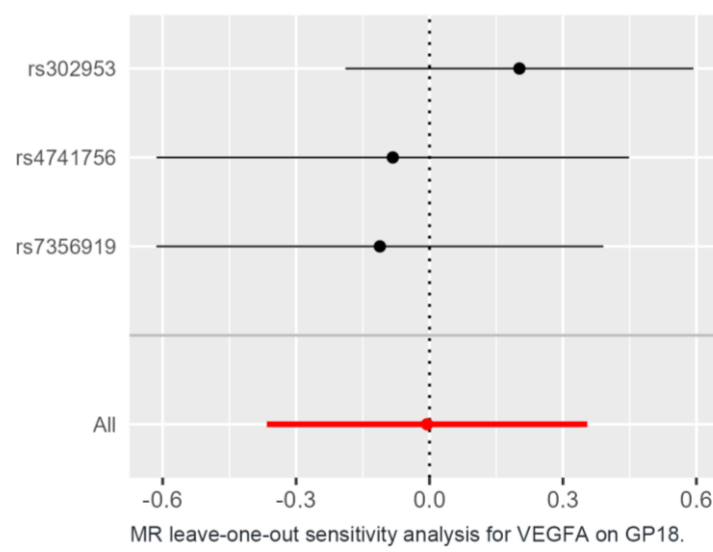**R**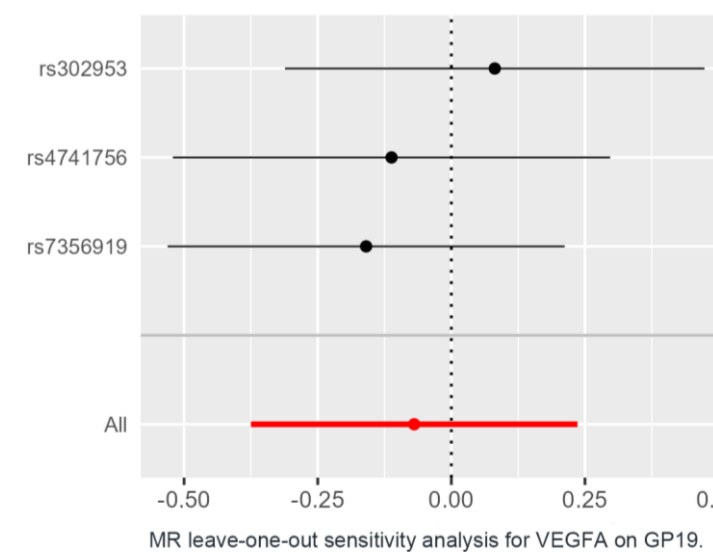**S**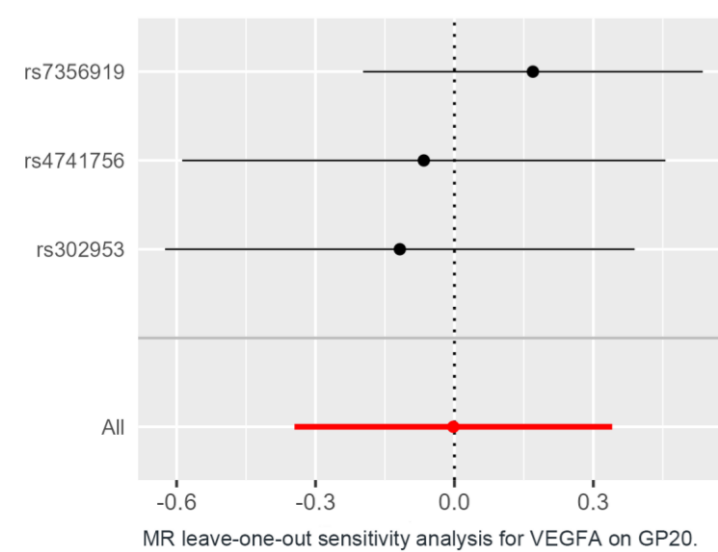**T**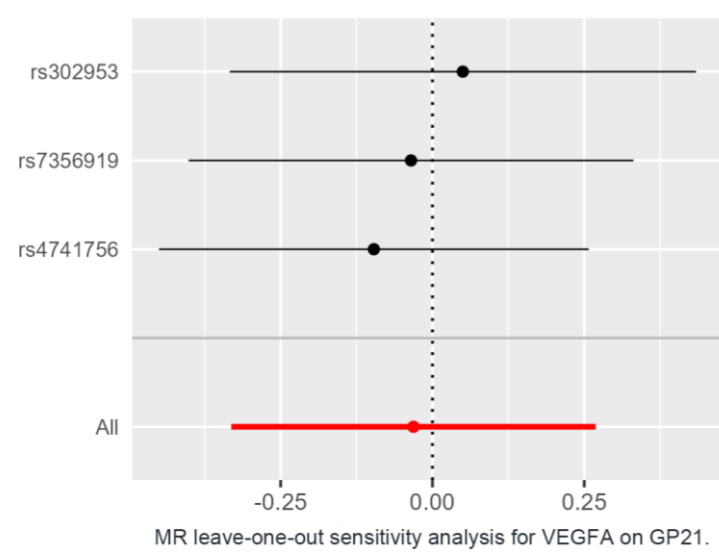**U**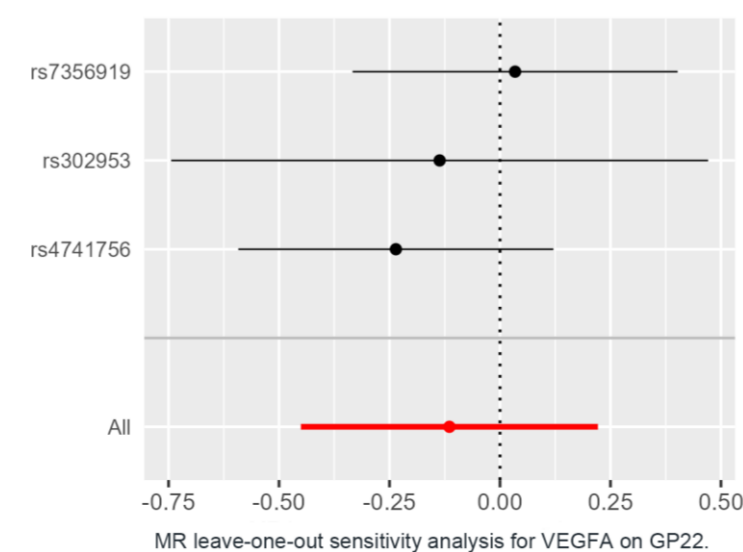**V**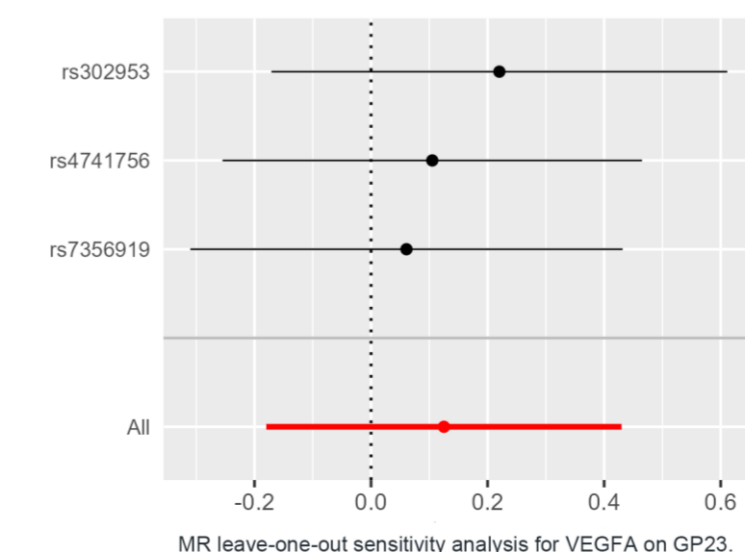**W**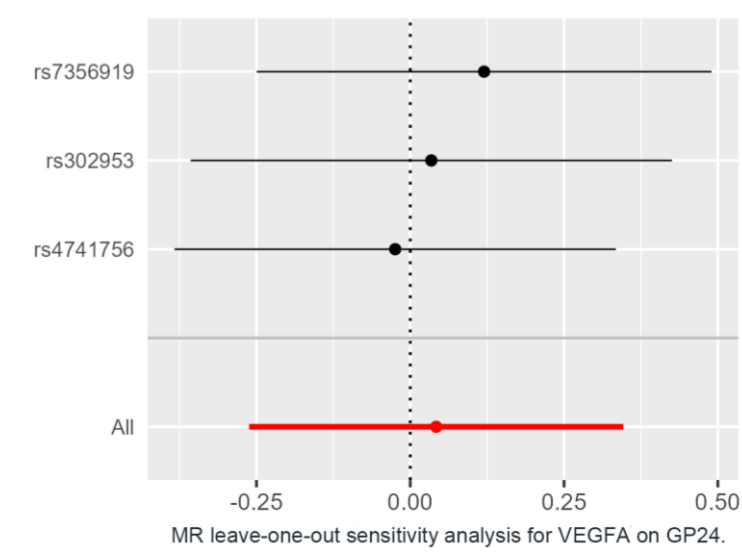

Supplement: Supplementary file 1 [file ijms-25-06337-s001.zip › Fig.S6-reverse-VEGFA.pdf]
